# MCM8IP activates the MCM8-9 helicase to promote DNA synthesis and homologous recombination upon DNA damage

**DOI:** 10.1038/s41467-020-16718-3

**Published:** 2020-06-11

**Authors:** Jen-Wei Huang, Ananya Acharya, Angelo Taglialatela, Tarun S. Nambiar, Raquel Cuella-Martin, Giuseppe Leuzzi, Samuel B. Hayward, Sarah A. Joseph, Gregory J. Brunette, Roopesh Anand, Rajesh K. Soni, Nathan L. Clark, Kara A. Bernstein, Petr Cejka, Alberto Ciccia

**Affiliations:** 1 Department of Genetics and Development, Herbert Irving Comprehensive Cancer Center, Columbia University Irving Medical Center, New York, NY USA; 2 Institute for Research in Biomedicine, Faculty of Biomedical Sciences, Università della Svizzera italiana, Bellinzona, Switzerland; 3 Institute of Biochemistry, Department of Biology, ETH Zurich, Zurich, Switzerland; 4 Department of Microbiology and Molecular Genetics, University of Pittsburgh School of Medicine, Pittsburgh, PA USA; 5 Proteomics and Macromolecular Crystallography Shared Resource, Herbert Irving Comprehensive Cancer Center, Columbia University Irving Medical Center, New York, NY USA; 6 Department of Human Genetics, University of Utah, Salt Lake City, UT USA

**Keywords:** Genomic instability, DNA damage and repair

## Abstract

Homologous recombination (HR) mediates the error-free repair of DNA double-strand breaks to maintain genomic stability. Here we characterize C17orf53/MCM8IP, an OB-fold containing protein that binds ssDNA, as a DNA repair factor involved in HR. MCM8IP-deficient cells exhibit HR defects, especially in long-tract gene conversion, occurring downstream of RAD51 loading, consistent with a role for MCM8IP in HR-dependent DNA synthesis. Moreover, loss of MCM8IP confers cellular sensitivity to crosslinking agents and PARP inhibition. Importantly, we report that MCM8IP directly associates with MCM8-9, a helicase complex mutated in primary ovarian insufficiency, and RPA1. We additionally show that the interactions of MCM8IP with MCM8-9 and RPA facilitate HR and promote replication fork progression and cellular viability in response to treatment with crosslinking agents. Mechanistically, MCM8IP stimulates the helicase activity of MCM8-9. Collectively, our work identifies MCM8IP as a key regulator of MCM8-9-dependent DNA synthesis during DNA recombination and replication.

## Abstract (web summary)

Homologous recombination (HR) is an essential DNA repair pathway for genomic stability. Here the authors show that C17orf53/MCM8IP, an OB-fold containing protein, promotes HR through direct binding and activation of the MCM8-9 helicase complex.

## Introduction

The repair of DNA double-strand breaks (DSBs) by homologous recombination (HR) is critical for genomic stability and tumor suppression^1^. HR is initiated by the nucleolytic degradation of DSBs to reveal 3′-ended single-strand DNA (ssDNA) tails, which are stabilized by the ssDNA-binding complex replication protein A (RPA)^2^. The RAD51 recombinase is then loaded onto the resected ends to form a RAD51-ssDNA nucleoprotein filament that can invade a homologous DNA duplex, resulting in the formation of a D-loop structure^3^. Within the D-loop, the invading DNA strand then primes DNA synthesis, which is catalyzed by replicative DNA polymerases in the presence of proliferating cell nuclear antigen (PCNA) and RPA^4^. While the process of D-loop extension is dependent on the Pif1 helicase in yeast^5^, it remains unclear whether PIF1 and/or other DNA helicases promote DNA synthesis at D-loops in higher eukaryotes.

MCM8 and MCM9 are paralogs of the MCM2-7 replicative helicase^6^. Distinct from MCM2-7, MCM8 and MCM9 form a complex with putative DNA helicase activity that is not required for DNA replication initiation^7,8^. Instead, the MCM8-9 complex has been implicated in HR in both mitotic and meiotic cells^9–11^. Consistently, *Mcm8*- and *Mcm9*-null female mice are sterile^11,12^ and women carrying biallelic mutations in MCM8-9 exhibit primary ovarian insufficiency (POI), a genetic syndrome characterized by reduced reproductive lifespan^13,14^. Furthermore, as a consequence of their role in HR, MCM8- and MCM9-deficient cells are particularly sensitive to DNA crosslinking agents and poly(ADP-ribose) polymerase (PARP) inhibition^9,10,15^.

MCM8-9 has been implicated in several activities both early and late in HR. MCM8-9 physically interacts with and stimulates MRE11 in DSB resection^16^. MCM8-9 is also required for efficient loading of RAD51 onto DNA-damaged chromatin, potentially acting as a RAD51 mediator^9,11^. In addition, MCM8-9 has been implicated in HR steps downstream of RAD51 loading^10,17^, including DNA damage-induced DNA synthesis at acutely stalled replication forks, presumably the sites of one-ended DSBs, and at I-SceI-generated two-ended DSBs^17^. These observations raise the possibility that MCM8-9 can facilitate D-loop extension during recombination-associated DNA synthesis^17,18^.

While regulation of MCM8-9 by the Fanconi anemia (FA)/BRCA pathway has been demonstrated^10,17^, our knowledge of the physical interactors of MCM8-9 involved in HR modulation remains incomplete. In this study, we report the characterization of C17orf53/MCM8IP as an interactor of MCM8-9 that is recruited to sites of DNA damage in an RPA-dependent manner. Notably, MCM8IP-deficient cells exhibit HR defects downstream of RAD51 loading, especially in long-tract gene conversion, and loss of MCM8IP is associated with cellular sensitivity to cisplatin and PARP inhibition. In addition, MCM8IP-deficient cells exhibit slower replication fork progression and increased fork stalling in response to cisplatin. Mechanistically, we find that interactions of MCM8IP with RPA1 and MCM8-9 facilitate HR and replication fork progression and promote chemoresistance. Furthermore, we demonstrate that MCM8IP stimulates the helicase activity of MCM8-9 in vitro. Collectively, our findings suggest a role for the MCM8IP–MCM8-9 complex in promoting DNA damage-associated DNA synthesis.

## Results

### C17orf53/MCM8IP is an RPA-associated factor

**Fig. 1:**
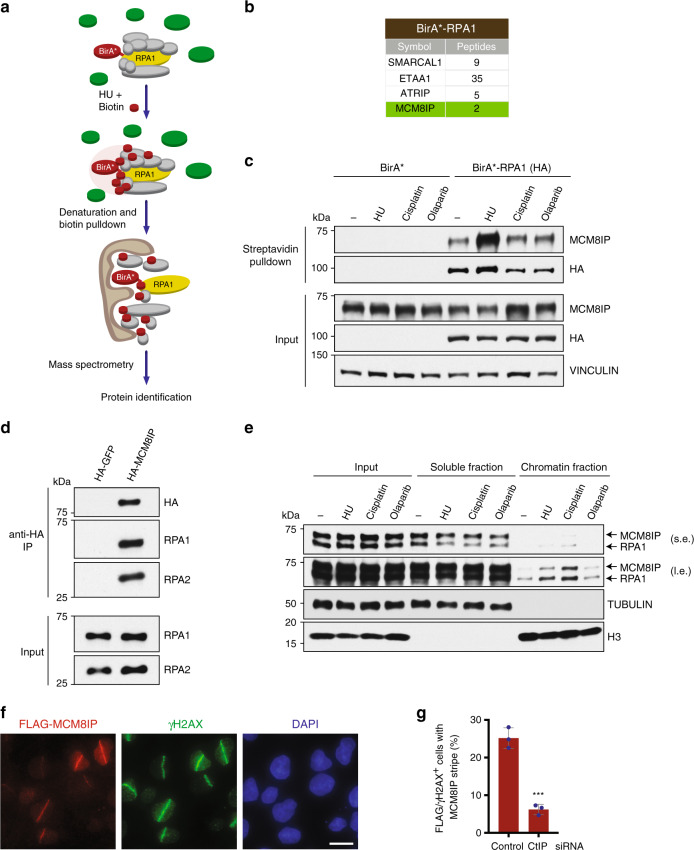
Identification of MCM8IP by proximity-dependent biotin-identification (BioID) technology. **a** Schematic of the protocol used to identify RPA1 interactors by BioID. HEK293T T-REx cells expressing doxycycline-inducible BirA*- or BirA*-RPA1 were treated with HU in the presence of exogenous biotin. Biotinylated proteins were captured in denaturing conditions from cell lysates by streptavidin pulldown and subjected to mass spectrometry for protein identification. **b** List of selected proteins identified by mass spectrometry that were enriched in streptavidin pulldowns conducted from BirA*-RPA1-expressing HEK293T T-REx cells relative to pulldowns performed from control BirA*-expressing cells. See also Supplementary Data 1. **c** Detection by western blot of MCM8IP in streptavidin pulldowns from HEK293T T-REx cells expressing BirA* or BirA*-RPA1. Cells were treated with HU (1 mM), cisplatin (20 µM), or olaparib (20 µM) in the presence of exogenous biotin for 24 h prior to lysis. Vinculin is shown as a loading control. **d** Detection by western blot of RPA1 and RPA2 following immunoprecipitation of HA-GFP or HA-MCM8IP from HEK293T cells. **e** Detection by western blot of RPA1 and MCM8IP (short exposure, s.e.; long exposure, l.e.) following subcellular fractionation of HCT116 cell lysates upon treatment with HU (1 mM), cisplatin (10 µM), or olaparib (10 µM) for 24 h. Tubulin and histone H3 are shown as loading and fractionation controls. **f** Representative images of FLAG-MCM8IP recruitment to sites of UV laser microirradiation in U2OS cells. DNA damage tracts are indicated with γH2AX staining. Scale bar = 20 µm. **g** Graphical representation of the percentage of FLAG-MCM8IP co-localizing with γH2AX following UV laser microirradiation in U2OS cells transfected with control or CtIP siRNA. The mean values ± SD of three independent experiments are presented. Statistical analysis relative to control siRNA was conducted using Student’s *t*-test (***p < 0.001, two-tailed).

Genome stability is maintained by a complex network of factors involved in DNA damage signaling, DNA replication, recombination, and repair^19^. To identify regulators of genome stability, we utilized the proximity-dependent biotin-identification (BioID) technology^20^. This approach employs a mutant isoform of the *Escherichia coli* biotin ligase BirA (BirA*) that exhibits promiscuous biotin ligase activity towards all proteins in close proximity, allowing the identification of both stable and transient protein–protein interactions. For these studies, we focused our attention on RPA, a central component of the DNA damage response that interacts with a multitude of DNA replication, recombination, and repair proteins^19,21^. To identify RPA-associated factors, we fused BirA* to the N-terminus of RPA1, the large subunit of the RPA trimer (Fig. 1a). To determine whether the BirA*–RPA1 fusion is functional, we expressed BirA*–RPA1 in U2OS cells and examined its localization in response to DNA damage. Following UV laser microirradiation, BirA*–RPA1 readily accumulated along the damaged tracts marked by γH2AX staining, indicating that fusion with BirA* did not impair the ability of RPA1 to localize to sites of DNA damage (Supplementary Fig. 1a). Next, we expressed doxycycline-inducible BirA*–RPA1 or the BirA*-alone control in HEK293T T-REx cells and performed a small-scale pulldown of biotinylated proteins using streptavidin beads in the presence or absence of hydroxyurea (HU). HU generates DSBs in S-phase after a prolonged treatment period^22^, thus being compatible with the slow labeling kinetics of BioID^20^. Relative to BirA* alone, BirA*–RPA1 was able to capture interactions with known RPA1 partners, such as RPA2 and SMARCAL1^23–27^, which were further enhanced with HU treatment, indicating that the BirA* tag does not alter the interaction of RPA1 with its partners (Supplementary Fig. 1b).

Having demonstrated the functionality of BirA*–RPA1, we performed streptavidin pulldowns in HU-treated HEK293T T-REx cells expressing BirA* alone or the RPA1 fusion and identified proteins by mass spectrometry. After only considering proteins represented by two or more peptides and at least 3-fold enriched over the control, BirA*–RPA1 identified 305 putative interactors (Supplementary Data 1). Several known RPA-binding proteins were identified, including ETAA1^28–30^, ATRIP^31^, and SMARCAL1^23–27^ (Fig. 1b). Interestingly, BirA*–RPA1 captured an interaction with C17orf53, a protein previously identified in a proteomic study of RPA-associated factors^32^. We were able to confirm the enrichment of C17orf53, which we named MCM8IP based on our subsequent findings, in streptavidin pulldowns from cells expressing BirA*–RPA1 by western blotting, particularly after HU treatment (Fig. 1c). To further validate this interaction, we conducted immunoprecipitations from cells expressing HA-tagged MCM8IP or RPA1. As shown in Fig. 1d, HA-MCM8IP was able to co-immunoprecipitate RPA from HEK293T cell extracts. Importantly, HA-RPA1 reciprocally co-immunoprecipitated endogenous MCM8IP, further confirming that MCM8IP is an RPA-associated factor (Supplementary Fig. 1c). These studies demonstrated that MCM8IP is a bona fide RPA-interacting protein.

### MCM8IP is recruited to chromatin after DNA damage

As the interaction between MCM8IP and BirA*–RPA1 is enhanced after treatment with DNA damaging agents (Fig. 1c), we sought to determine whether MCM8IP can be recruited to sites of DNA damage. To this end, we examined bulk chromatin association of MCM8IP by subcellular fractionation after treatment with DNA damaging agents. As shown in Fig. 1e, MCM8IP was recruited to chromatin after DNA damage, especially upon treatment with HU or the crosslinking agent cisplatin. Interestingly, we noted a positive correlation between the relative amounts of chromatin-bound MCM8IP and RPA1 among the different treatments (Fig. 1e). Next, we expressed FLAG-MCM8IP in U2OS cells and examined its localization in response to DNA damage. Following UV laser microirradiation, we observed MCM8IP accumulation onto chromatin, where it co-localized with γH2AX (Fig. 1f). Interestingly, MCM8IP localization was impaired by depletion of the DSB resection-promoting factor CtIP, suggesting that it depends on the generation of 3′ ssDNA ends by DSB resection (Fig. 1g and Supplementary Fig. 1d, e). These findings suggest that MCM8IP accumulates at DNA damage sites containing ssDNA regions.

### MCM8IP directly interacts with RPA1

**Fig. 2:**
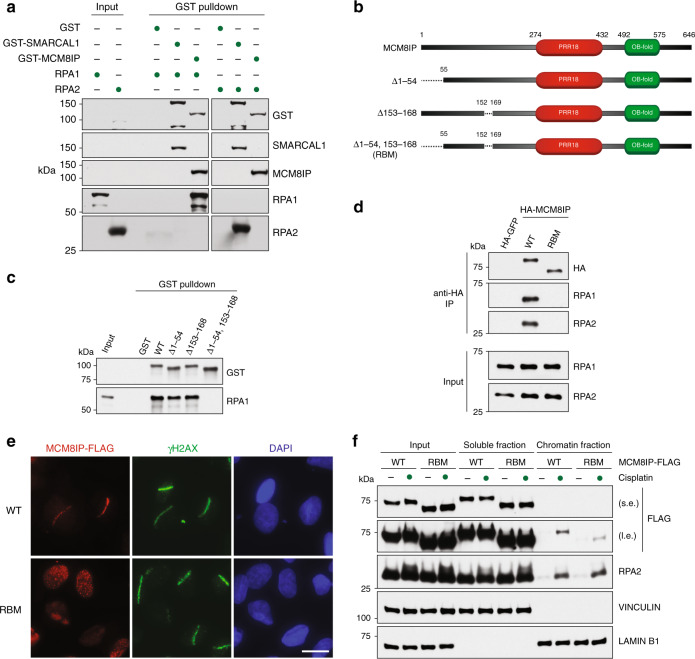
Characterization of the interaction between MCM8IP and RPA1. **a** Detection by western blot of RPA1 and RPA2 co-precipitated by bead-bound recombinant GST, GST-SMARCAL1, or GST-MCM8IP from bacteria. **b** Schematic representation of full-length MCM8IP and MCM8IP with deletions in RPA1 binding motifs. The Δ1–54,153–168 mutant was renamed RBM. Shown in red is a region with homology to proline-rich protein 18 (PRR18). The DUF4539 domain, a predicted OB-fold, is shown in green. **c** Detection by western blot of RPA1 co-precipitated by bead-bound GST, GST-MCM8IP WT, or GST fused to the MCM8IP mutants presented in (**b**). **d** Detection by western blot of RPA1 and RPA2 co-immunoprecipitated by HA-GFP, HA-MCM8IP WT, or RBM from HEK293T cells. **e** Representative images of the recruitment of MCM8IP-FLAG WT or RBM to sites of UV laser microirradiation in U2OS cells. DNA damage tracts are indicated with γH2AX staining. Scale bar = 20 µm. **f** Detection by western blot of MCM8IP-FLAG (short exposure, s.e.; long exposure, l.e.) and RPA2 following subcellular fractionation of lysates from HCT116 *MCM8IP* KO cells reconstituted with MCM8IP-FLAG WT or RBM. Cells were treated with 10 µM cisplatin for 24 h or left untreated prior to lysis. Vinculin and Lamin B1 are shown as loading and fractionation controls.

To determine whether MCM8IP and RPA1 interact directly, we partially purified GST-MCM8IP from bacteria and incubated it with lysates from bacteria expressing RPA1 or RPA2. As shown in Fig. 2a, GST-MCM8IP specifically co-precipitated RPA1, whereas our positive control, GST-SMARCAL1, co-precipitated RPA2, as previously reported^24^. To identify which region of MCM8IP binds RPA1, we constructed a series of deletion mutants of GST-MCM8IP and expressed them in bacteria (Supplementary Fig. 2a). Following GST pulldown of the above mutants, we identified a minimal region of MCM8IP containing the first 215 residues that was able to co-precipitate RPA1 (Supplementary Fig. 2b). Several proteins that interact with RPA1 do so through acidic residue-rich motifs, which engage the basic cleft of RPA1’s N-terminal OB-fold^28,33–36^. Through sequence analysis, we identified two conserved stretches of acidic residues within the N-terminal third of MCM8IP (Supplementary Fig. 2c). While deletion of either acidic motif from GST-MCM8IP mildly reduced the levels of co-precipitated RPA1 (Fig. 2b, c), a GST-MCM8IP mutant lacking both motifs (RBM, RPA1-Binding Mutant) displayed no detectable interaction with RPA1 (Fig. 2b, c). The deletion of both motifs also impaired the association of HA-MCM8IP with RPA in HEK293T cells, as determined by co-immunoprecipitation studies (Fig. 2d).

To determine whether the accumulation of MCM8IP at sites of DNA damage is dependent on its interaction with RPA, we subjected U2OS cells expressing MCM8IP-FLAG RBM to UV laser microirradiation. In these experiments, MCM8IP-FLAG RBM localized to the nucleus, but failed to accumulate at sites of UV microirradiation (Fig. 2e). Consistently, we also observed a reduction in the cisplatin-induced association of MCM8IP-FLAG RBM with bulk chromatin relative to WT MCM8IP-FLAG in HCT116 cells (Fig. 2f). Collectively, our findings indicate that MCM8IP contains two acidic motifs that mediate direct interaction with RPA1 and that this interaction is required for efficient MCM8IP recruitment to DNA-damaged chromatin.

### MCM8IP interacts with MCM8-9

**Fig. 3:**
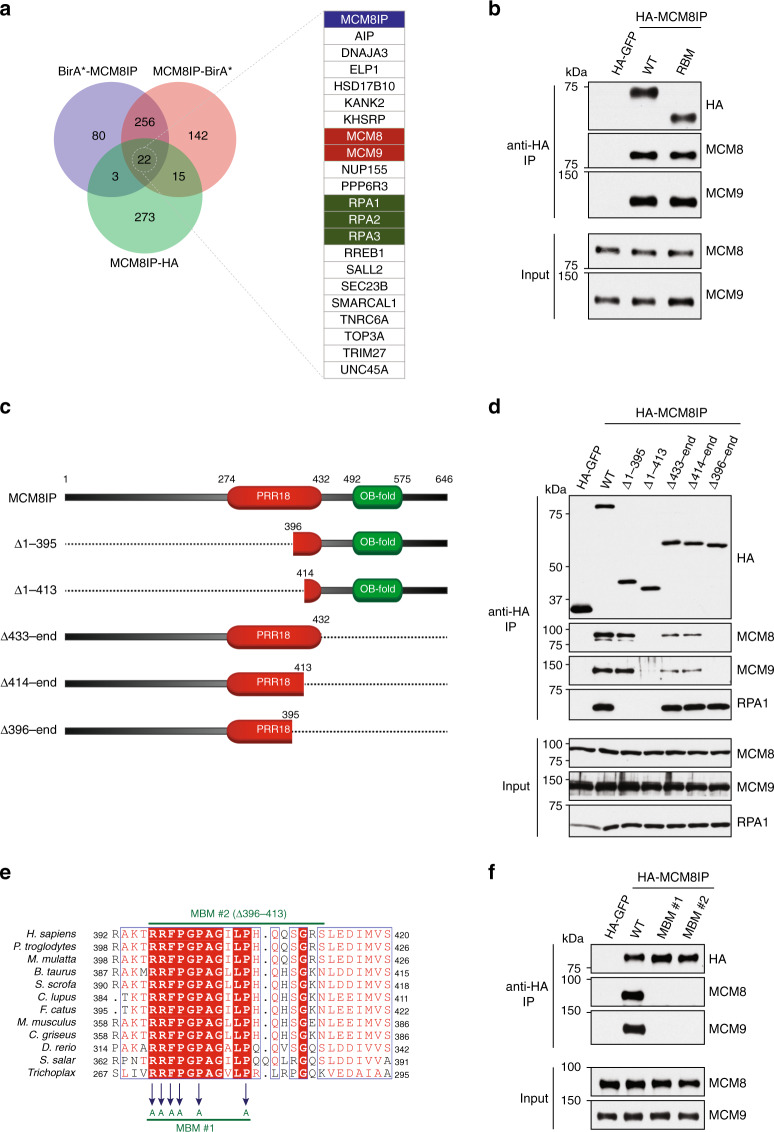
Characterization of the interaction between MCM8IP and MCM8-9. **a** Venn diagram of the number of proteins identified by mass spectrometry that were enriched in BirA*-MCM8IP and MCM8IP-BirA* streptavidin pulldowns from HEK293T T-REx cells or MCM8IP-HA immunoprecipitates from HEK293T cells relative to their respective controls. Proteins with multiple enriched isoforms were considered a single hit in this diagram. A list of proteins commonly identified by all three experiments is presented. See also Supplementary Data 2. **b** Detection by western blot of MCM8 and MCM9 co-immunoprecipitated by HA-GFP, HA-MCM8IP WT, or RBM from HEK293T cells. **c** Schematic representation of MCM8IP truncation mutants used to identify the region of interaction with MCM8-9. **d** Detection by western blot of MCM8, MCM9, and RPA1 co-immunoprecipitated from HEK293T cells by HA-GFP, HA-MCM8IP WT, and mutants presented in (**c**). **e** Alignment from various species of the minimal region of human MCM8IP required for MCM8-9 interaction. MCM8IP MBM #1 mutant carries alanine substitutions of the indicated residues. MCM8IP MBM #2 mutant carries a deletion of the indicated 18 amino acid residues. Sequence alignments were conducted using Clustal Omega and processed using ESPript. **f** Detection by western blot of MCM8 and MCM9 co-immunoprecipitated by HA-GFP, HA-MCM8IP WT, MBM #1 or MBM #2 from HEK293T cells.

Although MCM8IP contains no apparent catalytic domains, it does harbor a nucleic acid-binding OB-fold motif and a central region with homology to proline-rich protein 18 (PRR18), a protein of unknown function (Fig. 2b). To ascertain whether MCM8IP associates with other factors implicated in the DNA damage response, we performed streptavidin pulldowns from HU-treated HEK293T T-REx cells expressing doxycycline-inducible MCM8IP constructs with N- or C-terminal BirA* tags or co-immunoprecipitation from MCM8IP-HA-expressing HEK293T cells. By mass spectrometric analyses, we identified a set of 22 proteins commonly found in MCM8IP BioID and co-immunoprecipitation experiments that were represented by two or more unique peptides and were at least 5-fold enriched in total spectral counts relative to the respective controls (Fig. 3a and Supplementary Data 2). This set included all three subunits of RPA, as well as the RPA2 interactor SMARCAL1. MCM8 and MCM9 were also identified among these proteins (Fig. 3a), and their interaction with MCM8IP was validated by co-immunoprecipitation analyses in HEK293T cells (Fig. 3b and Supplementary Fig. 3a). Importantly, the MCM8IP RBM mutant co-immunoprecipitated comparable levels of MCM8-9 as WT MCM8IP, indicating that the MCM8IP–MCM8-9 interaction is independent of RPA1 binding (Fig. 3b).

Using N- and C-terminal truncation mutants of MCM8IP (Fig. 3c), we identified a region spanning residues 396–413 that was sufficient for MCM8-9 interaction (Fig. 3d). This region of MCM8IP contains several residues that are conserved among metazoans (Fig. 3e). Substitution of six of the conserved residues with alanines (MCM8-9 Binding Mutant #1, MBM #1) or deletion of the entire region (MBM #2) led to abrogation of the interaction of MCM8IP with MCM8-9 (Fig. 3e, f). Consistently, we observed that recombinant MCM8IP interacts directly with MCM8, either alone or in complex with MCM9, and this interaction is disrupted in the MBM #1 mutant (Supplementary Fig. 3b, c). In further support of an evolutionarily conserved interaction between MCM8IP and MCM8-9, we find that the *MCM8IP*, *MCM8*, and *MCM9* genes significantly co-occur among multicellular eukaryotes (*p*-values: 3.48 × 10^−5^ for MCM8–MCM9; 8.61 × 10^−5^ for MCM8–MCM8IP; 9.16 × 10^−4^ for MCM9–MCM8IP), suggesting that MCM8IP is dependent on MCM8-9 to perform its function (Supplementary Fig. 3d and Supplementary Data 3 and 4).

To determine whether the recruitment of MCM8IP to sites of DNA damage is dependent on its association with MCM8-9, U2OS cells expressing MCM8IP-FLAG MBM #2 were subjected to UV laser microirradiation. As shown in Supplementary Fig. 4a, b, MCM8IP-FLAG MBM #2 was recruited to sites of UV laser microirradiation with efficiency similar to WT MCM8IP. Additionally, we observed that WT MCM8IP-FLAG was able to form nuclear foci following cisplatin treatment in HCT116 cells (Supplementary Fig. 4c–e). Consistent with our UV laser microirradiation experiments, MCM8IP-FLAG MBM #2, but not RBM, was as proficient as WT MCM8IP-FLAG for cisplatin-induced foci formation (Supplementary Fig. 4c–e). Collectively, these results indicate that interaction with MCM8-9 is not required for MCM8IP recruitment to sites of DNA damage.

### The MCM8IP–MCM8-9 complex interacts with ssDNA

Our previous observations suggest that MCM8IP associates with ssDNA-containing regions in mammalian cells (Fig. 1g and Supplementary Fig. 1d, e). To determine whether MCM8IP directly binds ssDNA, we purified FLAG-tagged MCM8IP (Supplementary Fig. 5a) from insect cells and incubated it with ^32^P-labeled ssDNA. As shown in Supplementary Fig. 5d, e, MCM8IP associates with ssDNA at higher concentrations (≥32 nM). Likewise, recombinant MCM8-9 (20 nM) purified from insect cells (Supplementary Fig. 5c) also exhibits ssDNA-binding activity (Supplementary Fig. 5f, lane b in both panels). To determine whether the MCM8IP–MCM8-9 complex also binds ssDNA, we incubated ssDNA with MCM8-9 (20 nM) and either the WT or MBM #1 mutant form of MCM8IP (20 nM). As shown in Supplementary Fig. 5f, the addition of WT MCM8IP, but not MBM #1 mutant protein (Supplementary Fig. 5b, left panel), led to the formation of protein–ssDNA complexes with slower electrophoretic migration relative to ssDNA complexes containing MCM8-9 alone (Supplementary Fig. 5f, compare lanes d with b and f, both panels). These data indicate that MCM8IP and MCM8-9 bind ssDNA as a protein complex.

**Fig. 4:**
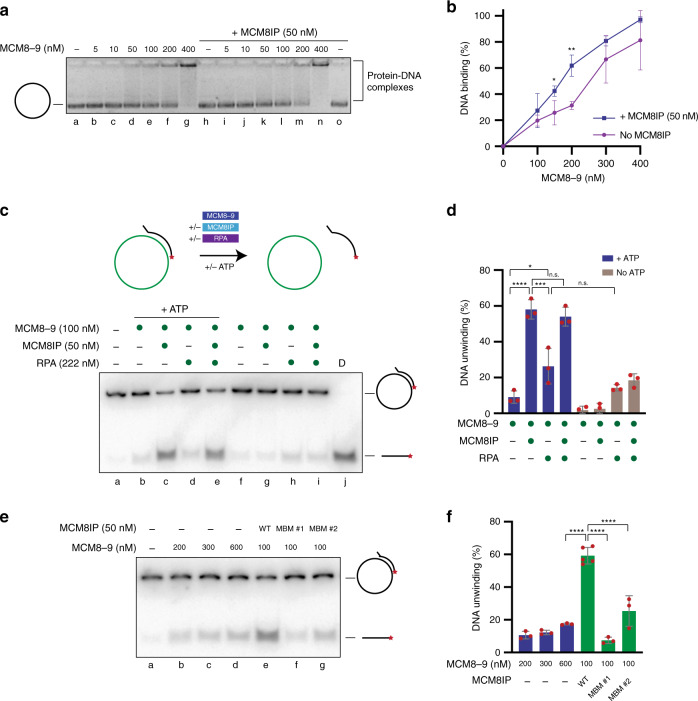
Analysis of the DNA binding and helicase activities exhibited by MCM8-9 and MCM8IP. **a** Representative gel of an electrophoretic mobility shift assay with a ssDNA plasmid (M13mp18, 100 ng per reaction) incubated with increasing amounts of recombinant MCM8-9 in the presence or absence of MCM8IP (50 nM). **b** Graphical representation of the percentage of ssDNA plasmid bound by recombinant MCM8-9 in the presence or absence of MCM8IP in electrophoretic mobility shift assays conducted as in (**a**). The mean ± SD of three or more independent experiments (*n* = 3–4) is presented. Statistical analysis was conducted using Student’s *t*-test (*p < 0.05, **p < 0.01, two-tailed). **c** Schematics of a DNA unwinding reaction conducted using a ^32^P-labeled ssDNA oligo annealed to a ssDNA plasmid (M13mp18) in the presence of the indicated proteins (top panel). Representative autoradiograph of a DNA unwinding reaction conducted using the depicted DNA substrate in the presence or absence of ATP with purified MCM8-9 (100 nM), WT MCM8IP (50 nM), or RPA (222 nM), either alone or in combination (bottom panel). D—boiled DNA substrate control. **d** Graphical representation of the percentage of DNA unwinding in reactions conducted as in (**c**). The mean ± SD of three independent experiments is presented. Statistical analysis was conducted using one-way ANOVA (*p < 0.05, ***p < 0.001, ****p < 0.0001). **e** Representative autoradiograph of the ATP-dependent DNA unwinding reaction shown in (**c**) conducted with increasing concentrations of MCM8-9 alone (lanes b–d) or at a fixed MCM8-9 concentration (100 nM) in the presence of WT MCM8IP, MBM #1 or MBM #2 proteins (each at 50 nM, lanes e–g). **f** Graphical representation of the percentage of DNA unwinding in reactions conducted as in (**e**) in the presence of ATP. The mean ± SD of at least three independent experiments (*n* = 3–5) is presented. Statistical analysis was conducted as in (**d**) (****p < 0.0001).

Next, we sought to determine whether MCM8IP modulates the affinity of MCM8-9 for ssDNA. To this end, we quantified the binding of MCM8-9 to a ssDNA plasmid in the presence or absence of WT MCM8IP (Fig. 4a, b). Incubation with WT MCM8IP protein at a concentration where it does not detectably bind ssDNA (Fig. 4a, lane h) led to a modest but statistically significant increase in ssDNA binding by MCM8-9 (Fig. 4a, compare lane m with f, and Fig. 4b). Collectively, these results indicate that MCM8IP and MCM8-9 are ssDNA-binding proteins that exhibit enhanced ssDNA-binding activity as a complex.

### MCM8IP stimulates the helicase activity of MCM8-9

Recombinant MCM8 and purified MCM9-containing complexes from HeLa cells exhibit helicase activity in vitro^7,37^. We therefore sought to determine whether MCM8IP may regulate MCM8-9 helicase activity. In these studies, we noted that purified MCM8-9 alone exhibited limited ability to unwind a plasmid-based substrate previously used to assay MCM helicase activity^37,38^ (Fig. 4c, compare lane b with a). Remarkably, WT MCM8IP led to ~6-fold stimulation of the helicase activity of MCM8-9 (Fig. 4c, compare lane c with b, and Fig. 4d), while no substrate unwinding was observed in the presence of MCM8IP alone (Supplementary Fig. 5g). Interestingly, RPA did not further promote DNA unwinding by MCM8IP and MCM8-9 (Fig. 4c, compare lane e with c, and Fig. 4d). The limited strand separation observed in RPA-containing reactions in the absence of ATP (Fig. 4c, lanes h and i) is the likely result of the known DNA melting activity of RPA^39^. Furthermore, MBM #1 or MBM #2 mutant proteins (Supplementary Fig. 5b) did not significantly stimulate MCM8-9-dependent DNA unwinding, as compared to WT MCM8IP protein (Fig. 4e, compare lanes f and g with e, and Fig. 4f). Collectively, these experiments indicate that MCM8IP, through its MCM8-9 binding region, specifically stimulates the helicase activity of MCM8-9.

### MCM8IP regulates homologous recombination

**Fig. 5:**
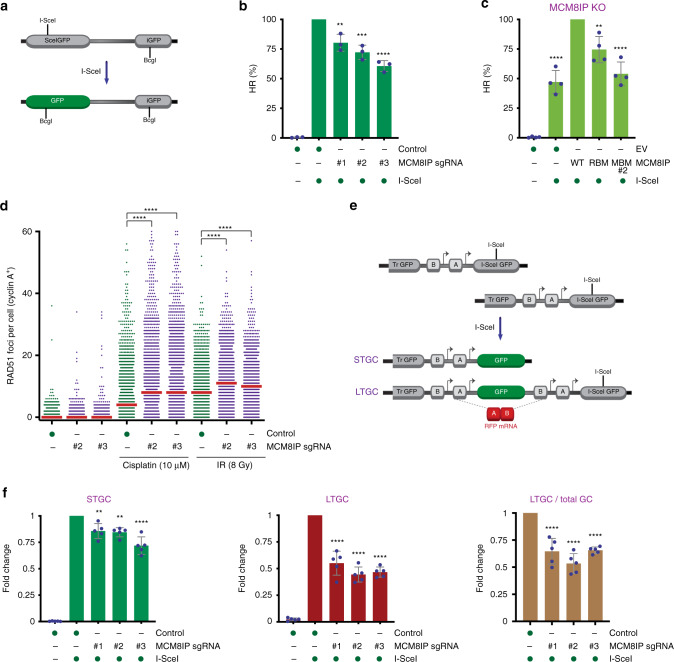
Homologous recombination frequency in MCM8IP-deficient cells. **a** Schematic representation of the DR-GFP reporter of gene conversion, as described^80^. **b** Graphical representation of the percentage of I-SceI-induced HR events in U2OS DR-GFP cells expressing the indicated MCM8IP sgRNAs relative to control cells. The relative level of background events in control cells transfected with an empty vector is additionally shown. The mean ± SD of three independent experiments is presented. Statistical analysis relative to the control was conducted using one-way ANOVA (**p < 0.01, ***p < 0.001, ****p < 0.0001). **c** Graphical representation of the percentage of I-SceI-induced HR events in an U2OS DR-GFP *MCM8IP* KO clone reconstituted with the indicated cDNAs or an empty vector (EV) control, as shown in Supplementary Fig. 6d, e, relative to WT MCM8IP-expressing KO cells. The relative level of background events in EV-complemented cells transfected with an empty vector is additionally shown. The mean ± SD of four independent experiments is presented. Statistical analysis relative to WT MCM8IP-expressing KO cells was conducted as in (**b**) (**p < 0.01, ****p < 0.0001). **d** Dot plot of the number of cisplatin- or ionizing radiation-induced RAD51 foci in cyclin A-positive HCT116 control cells or cells expressing the indicated MCM8IP sgRNAs. The median values are indicated by red lines within the dot plot. Data are representative of two independent experiments. Statistical analysis was conducted using a Mann–Whitney test (****p < 0.0001, two-tailed). **e** Schematic representation of the SCR/RFP reporter of gene conversion, as described^44^. Blocks A and B are artificial *RFP* exons and arrows are promoters. Short-tract gene conversion (STGC) results in GFP expression alone, while long-tract gene conversion (LTGC) results in the additional expression of RFP. **f** Graphical representation of the fold change in I-SceI-induced STGC (left panel), LTGC (middle panel), and LTGC as a fraction of total gene conversion events (right panel) in U2OS 35S cells expressing the indicated MCM8IP sgRNAs relative to control cells. Relative levels of background STGC and LTGC events in U2OS 35S control cells transfected with an empty vector are additionally shown. The mean ± SD of five independent experiments is presented. Statistical analysis was conducted as in (**b**) (**p < 0.01, ****p < 0.0001).

Since *MCM8IP* co-evolved significantly with genes involved in the FA and HR repair pathways (Supplementary Fig. 6a), we sought to determine whether MCM8IP regulates HR. To this end, we targeted *MCM8IP* with three different sgRNAs using CRISPR-Cas9 in U2OS cells carrying the HR reporter construct DR-GFP (Fig. 5a and Supplementary Fig. 6b)^40^. Following induction of an I-SceI-mediated DSB in DR-GFP, we observed reductions in the efficiency of gene conversion, as determined by measuring the percentage of GFP-positive cells (Fig. 5b and Supplementary Fig. 6c). To rule out off-target effects of Cas9/sgRNA editing, we derived a clone from U2OS DR-GFP cells expressing MCM8IP sgRNA #3 (Supplementary Fig. 6d). Expression of MCM8IP WT cDNA in the *MCM8IP* KO clone significantly increased HR efficiency relative to the empty vector control, indicating that the HR defects observed were due to specific targeting of *MCM8IP* (Fig. 5c and Supplementary Fig. 6e, f). Interestingly, neither expression of MCM8IP RBM nor MBM cDNA in the *MCM8IP* KO clone were able to restore HR efficiency to that of WT cDNA, suggesting that interactions with RPA1 and MCM8-9 are required for efficient MCM8IP-dependent HR (Fig. 5c and Supplementary Fig. 6e). Impaired HR activity was also observed when *MCM8IP* was disrupted with three different sgRNAs in HEK293T cells carrying a BFP-based reporter of Cas9-mediated HR (Supplementary Fig. 7a–d)^41^, which can be converted into GFP upon Cas9-mediated cleavage and recombination with a dsDNA donor template carrying a single nucleotide substitution (c.197C>T, p.His66Tyr)^42,43^. These studies further confirmed the requirement of MCM8IP for HR.

To determine the underlying HR defect in MCM8IP-deficient cells, we examined the efficiency of DNA damage-induced RAD51 foci formation upon MCM8IP loss. Interestingly, MCM8IP deficiency led to increased accumulation of RAD51 foci in HCT116 cells in response to either cisplatin or ionizing radiation (Fig. 5d). The increase in RAD51 foci observed in MCM8IP-deficient cells was not accompanied by elevated RPA foci formation after cisplatin treatment (Supplementary Fig. 7e), suggesting that MCM8IP-deficient cells do not exhibit enhanced formation of RPA-coated ssDNA. These observations suggest that the HR defect exhibited by MCM8IP-deficient cells occurs downstream of RAD51 nucleofilament formation or RAD51-mediated strand invasion.

MCM8-9 has been implicated in DNA synthesis downstream of RAD51 loading at collapsed replication forks and I-SceI-induced DSBs^17^. To investigate the possible role of MCM8IP in HR steps following RAD51-mediated strand invasion, we evaluated the effect of MCM8IP loss on long-tract gene conversion (LTGC), which represents a measure of recombination-associated DNA synthesis^17^. To this end, we targeted MCM8IP in 35S cells, a line of U2OS cells that carries the SCR/RFP reporter^44^. Following I-SceI-induced DSB formation in an inactive GFP gene, LTGC events involving 1.3–3.4 kb of nascent DNA synthesis result in the restoration of the GFP open reading frame and duplication of an RFP cassette, leading to RFP expression (Fig. 5e). LTGC can therefore be evaluated by the percentage of GFP/RFP double-positive cells. Consistent with the finding from U2OS DR-GFP cells, we observed a general decrease in gene conversion in U2OS 35S cells upon MCM8IP loss (Fig. 5f and Supplementary Fig. 7f, g). Notably, however, MCM8IP loss led to a reduction of LTGC events among total GC events, indicating that MCM8IP is primarily needed for GC events that require extensive DNA synthesis. Collectively, these findings suggest that MCM8IP can promote DSB repair through regulation of HR-associated DNA synthesis.

### MCM8IP mediates cellular resistance to DNA damaging agents

**Fig. 6:**
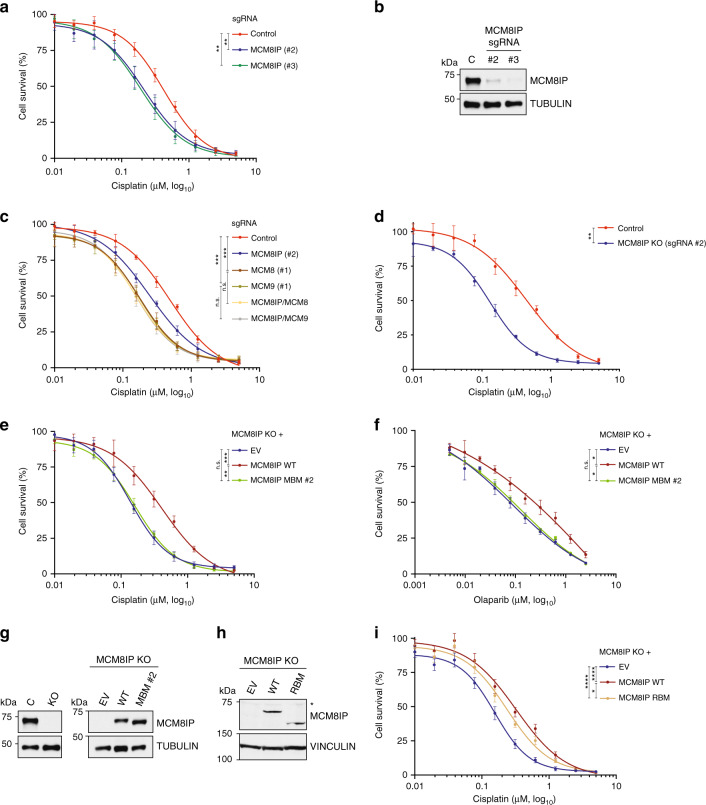
Survival analysis in MCM8IP-deficient cells treated with cisplatin or olaparib. **a** Survival analysis in HCT116 control cells or cells expressing the indicated MCM8IP sgRNAs upon treatment with cisplatin. Cell survival is expressed as a percentage of an untreated control. The mean ± SD of three independent experiments is presented. Statistical analysis was conducted on data points at three distinct cisplatin concentrations (0.625, 0.313, 0.156 µM) using Student’s *t*-test (**p < 0.01, at all three concentrations, two-tailed). **b** Detection by western blot of MCM8IP in HCT116 control cells or cells expressing the indicated MCM8IP sgRNAs utilized in the assay shown in (**a**). Tubulin is shown as a loading control. **c** Survival analysis of cisplatin-treated HCT116 control cells or cells expressing the indicated sgRNAs. Cell survival is shown as in (**a**). The mean ± SD of three or more independent experiments (*n* = 3–4) is presented. Statistical analysis was conducted as in (**a**) (***p < 0.001, at all three concentrations analyzed). **d** Survival analysis of HCT116 control cells or an HCT116 *MCM8IP* KO clone in response to cisplatin. Cell survival is represented as in (**a**). The mean ± SD of three independent experiments is presented. Statistical analysis was conducted as in (**a**) (**p < 0.01, at all three concentrations analyzed). **e** Survival analysis in the HCT116 *MCM8IP* KO clone shown in (**g**) reconstituted with MCM8IP WT, MBM #2, or an empty vector (EV) control in response to cisplatin. Cell survival is shown as in (**a**). The mean ± SD of four independent experiments is presented. Statistical analysis was conducted as in (**a**) (**p < 0.01, ***p < 0.001, at all three concentrations analyzed). **f** Survival analysis in the HCT116 *MCM8IP* KO clone shown in (**g**) reconstituted with MCM8IP WT, MBM #2, or an EV control in response to olaparib. Cell survival is represented as in (**a**). The mean ± SD of three independent experiments is presented. Statistical analysis was conducted on data points at three distinct olaparib concentrations (0.313, 0.156, 0.078 µM) as in (**a**) (*p < 0.05, at all three concentrations). **g** Detection by western blot of MCM8IP in HCT116 control cells or in an *MCM8IP* KO clone (left panel), and in the same *MCM8IP* KO clone reconstituted with MCM8IP WT, MBM #2, or an EV control (right panel). Tubulin is shown as a loading control. **h** Detection by western blot of MCM8IP in an HCT116 *MCM8IP* KO clone reconstituted with MCM8IP WT, RBM, or an EV control. Vinculin is shown as a loading control. Asterisk indicates a non-specific band. **i** Survival analysis in the HCT116 *MCM8IP* KO clone shown in (**h**) reconstituted with MCM8IP WT, RBM, or an EV control in response to cisplatin. Cell survival is shown as in (**a**). The mean ± SD of nine independent experiments is presented. Statistical analysis was conducted as in (**a**) (*p < 0.05, ****p < 0.0001, at all three concentrations analyzed).

A hallmark of HR-deficient cells is hypersensitivity to DNA crosslinking agents^45^. We therefore sought to determine whether the HR defects of MCM8IP-deficient cells are associated with a vulnerability to cisplatin-induced cell death. To this end, we subjected HCT116 cells expressing MCM8IP sgRNAs (#2 and #3) to varying doses of cisplatin (Fig. 6a, b). As shown in Fig. 6a, we observed a notable reduction in cell survival of MCM8IP-deficient cells following cisplatin treatment relative to the control. These results indicate that MCM8IP-deficient cells exhibit enhanced sensitivity to treatment with crosslinking agents.

MCM8-9 deficiency is characterized by sensitivity to crosslinking agents in various species^9–11^, raising the possibility that MCM8IP cooperates with MCM8-9 to promote chemoresistance. We therefore examined whether MCM8IP and MCM8-9 genetically interact. To this end, we targeted MCM8 or MCM9 with sgRNAs in HCT116 control cells or cells expressing MCM8IP sgRNAs (#2 and #3) (Supplementary Fig. 8a). As expected, loss of either MCM8 or MCM9 significantly sensitized control HCT116 cells to cisplatin (Fig. 6c and Supplementary Fig. 8b). Importantly, the loss of MCM8 or MCM9 did not further sensitize MCM8IP-deficient cells to cisplatin treatment (Fig. 6c and Supplementary Fig. 8b). These results suggest an epistatic relationship between MCM8IP and MCM8-9 with regard to cellular resistance to cisplatin.

Next, we sought to determine which interacting regions of MCM8IP were required for chemoresistance. To this end, we derived an *MCM8IP* KO clone from HCT116 cells expressing sgRNA #2 and confirmed its sensitivity to cisplatin relative to control cells (Fig. 6d, g). *MCM8IP* KO cells also exhibited sensitivity to the PARP inhibitor olaparib (Supplementary Fig. 8c), in line with the previously observed sensitivity of MCM8/9-deficient cells to PARP inhibition^15^. Interestingly, expression of WT MCM8IP, but not MBM #1 or #2 mutant, cDNAs in *MCM8IP* KO cells complemented chemoresistance relative to empty vector control cells (Fig. 6e–g and Supplementary Fig. 8d, e). Additionally, the expression of MCM8IP RBM cDNA did not fully complement chemoresistance as compared to WT MCM8IP-expressing KO cells (Fig. 6h, i). These results suggest that interactions with MCM8-9 and RPA1 are required for MCM8IP-dependent cellular resistance to DNA damaging agents.

### MCM8IP promotes DNA synthesis in response to DNA damage

**Fig. 7:**
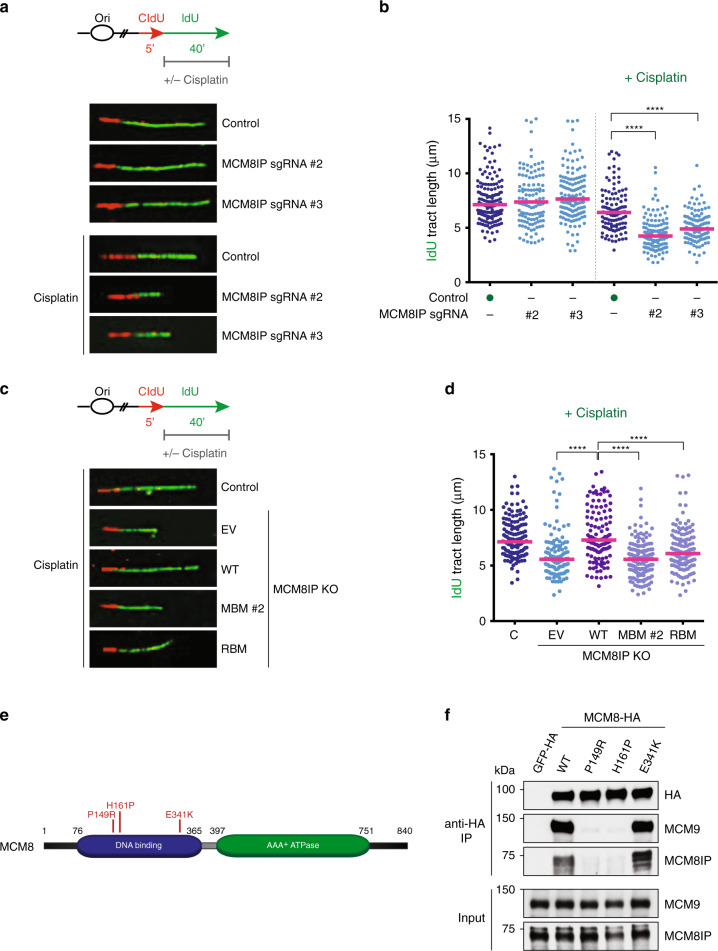
Analysis of replication fork progression in MCM8IP-deficient cells in response to cisplatin treatment. **a** Schematic representation of the CldU/IdU pulse-labeling assay (top panel). IdU labeling was performed in the absence or presence of cisplatin (30 µM). Representative images of fibers analyzed in untreated or cisplatin-treated HCT116 control cells or cells expressing the indicated MCM8IP sgRNAs (bottom panel). **b** Dot plot of IdU tract length for individual replication forks in untreated or cisplatin-treated HCT116 control cells or cells expressing the indicated MCM8IP sgRNAs. Experiments were conducted as shown in (**a**). The median values are indicated by red lines. Statistical analysis was conducted using a Mann–Whitney test (****p < 0.0001, two-tailed). Data are representative of two independent experiments. **c** Schematic representation of the CldU/IdU pulse-labeling assay as in (**a**, top panel). Representative images of fibers analyzed in cisplatin-treated HCT116 control cells or *MCM8IP* KO cells reconstituted with MCM8IP WT, MBM #2, RBM, or an empty vector (EV) control (bottom panel), as shown in Supplementary Fig. 9c. **d** Dot plot of IdU tract length for individual replication forks in cisplatin-treated HCT116 control cells or *MCM8IP* KO cells reconstituted with MCM8IP WT, MBM #2, RBM, or EV. Experiments were conducted as shown in (**c**). Data are shown and analyzed using a Mann–Whitney test as in (**b**) (****p < 0.0001, two-tailed), and are representative of two independent experiments. **e** Schematic representation of MCM8 with POI-associated mutations indicated. The DNA binding domain is indicated in blue and the ATPase domain is indicated in green. **f** Detection by western blot of MCM8IP and MCM9 co-immunoprecipitated from HEK293T cells by GFP-HA, MCM8-HA WT, or carrying the POI-associated mutations indicated in (**e**).

DNA crosslinks can result in replication fork arrest^46,47^. Given the hypersensitivity of MCM8IP-deficient cells to crosslinking agents, we examined whether MCM8IP deficiency affects replication fork progression upon cisplatin treatment using the DNA fiber assay (Fig. 7a). In untreated conditions, HCT116 cells expressing either control or MCM8IP sgRNAs exhibited similar rates of DNA synthesis, as indicated by the comparable lengths of IdU-labeled tracts (Fig. 7a, b). While the addition of cisplatin only mildly reduced fork progression in HCT116 control cells, cells expressing either MCM8IP sgRNA exhibited significantly shorter tracts of DNA synthesis (Fig. 7a, b). Consistently, we also observed increased cisplatin-induced fork stalling in MCM8IP-deficient cells (Supplementary Fig. 9a). Collectively, these results indicate that MCM8IP is required for efficient DNA synthesis in the presence of cisplatin.

We next examined which interacting regions of MCM8IP are required to maintain fork progression on cisplatin-damaged DNA by monitoring DNA synthesis in our HCT116 *MCM8IP* KO clone expressing either the WT, MBM #2 mutant, or RBM mutant cDNA of MCM8IP (Fig. 7c, d and Supplementary Fig. 9b, c). Consistent with our earlier results (Fig. 7b), replication fork progression was comparable between *MCM8IP* KO and control HCT116 cells in unperturbed conditions (Supplementary Fig. 9b). Importantly, the addition of cisplatin strongly impeded fork progression in *MCM8IP* KO cells relative to the control, and this effect was largely rescued by the expression of WT MCM8IP cDNA (Fig. 7d). Intriguingly, neither MBM #2 nor the RBM mutant was able to restore proper fork progression upon cisplatin treatment (Fig. 7d and Supplementary Fig. 9c). These findings suggest that interactions with both RPA1 and MCM8-9 are required for MCM8IP to maintain proper replication fork progression in the presence of cisplatin-damaged DNA.

### POI patient mutations disrupt the MCM8IP–MCM8 interaction

Biallelic mutations in *MCM8* predispose women to POI^14^. This phenotype is consistent with the sterility of female *Mcm8*-null mice, which exhibit defects in gametogenesis due to impaired HR during meiosis^11^. Having demonstrated the functional cooperation between MCM8IP and MCM8-9, we examined whether POI-associated MCM8 mutations (i.e., P149R, H161P, E341K, Fig. 7e)^48–51^ could disrupt the interaction of MCM8 with MCM8IP. To this end, we expressed either WT or mutant MCM8-HA in HEK293T cells and subjected the cell lysates to anti-HA immunoprecipitation. As shown in Fig. 7f, MCM8 carrying P149R or H161P mutations co-immunoprecipitated significantly less MCM8IP and MCM9 than the WT control or MCM8 E341K. These results indicate that disruption of the MCM8IP–MCM8–MCM9 complex may underlie the etiology of POI in patients carrying certain MCM8 mutations.

## Discussion

Our study characterizes the C17orf53 protein and its interactions with RPA1 and MCM8-9 in the regulation of DNA replication and repair in response to DNA damage. Accordingly, we refer to C17orf53 as MCM8IP (MCM8-9-Interacting Protein). We find that the HR defect caused by MCM8IP deficiency is consistent with an impairment in recombination-associated DNA synthesis downstream of RAD51 loading (Fig. 5d–f), as is the case with MCM8-9-deficient cells^17^. We also demonstrate that the MCM8IP–RPA1 and MCM8IP–MCM8-9 interactions facilitate HR and promote replication fork progression and cellular resistance upon treatment with DNA damaging agents (Figs. 5–7). Importantly, we report that MCM8IP increases the affinity of MCM8-9 for ssDNA and stimulates MCM8-9 helicase activity (Fig. 4). Collectively, these findings highlight the importance of MCM8IP in promoting DNA damage-associated DNA synthesis at replication and recombination intermediates. These observations are in line with the findings of a recent study on C17orf53 (renamed HROB by the authors) that was published during the revisions of our manuscript^52^.

Previous studies indicate a multistage role for MCM8-9 in promoting homology-directed repair of DSBs^16,17^, with its different functions potentially regulated through distinct and/or mutually exclusive binding partners. For example, MCM8-9 may regulate DSB resection early in HR upon association with the MRE11–RAD50–NBS1 complex^16^. Rather than a role in DSB resection or RAD51 loading (Fig. 5d and Supplementary Fig. 7e), we propose that the MCM8IP–MCM8-9 complex promotes recombination-associated DNA synthesis late in HR (Fig. 5f).

It has been proposed that RPA-coated D-loops may serve to localize the MCM8IP–MCM8-9 complex to sites of DNA synthesis during HR^52^. In agreement with those findings, we demonstrate that interaction with RPA1 is indeed required for optimal loading of MCM8IP onto damaged chromatin (Fig. 2e, f and Supplementary Fig. 4c, d). Interestingly, we observed residual chromatin loading of MCM8IP RBM mutant in response to DNA damage, albeit at lower levels compared to WT MCM8IP (Fig. 2f). This observation may explain the partial complementation of HR deficiency and cisplatin sensitivity observed in *MCM8IP* KO cells upon expression of the MCM8IP RBM mutant (Figs. 5c and 6i). In the absence of RPA1 interaction, MCM8IP may still be loaded onto damaged DNA by other means, such as through its ability to directly bind ssDNA (Supplementary Fig. 5d, e). Regardless of whether MCM8IP loading occurs as a consequence of RPA interaction and/or direct ssDNA binding, once on damaged chromatin MCM8IP may facilitate the localization of MCM8-9 at DNA repair sites, as previously suggested^52^, in line with our findings that MCM8IP increases the affinity of MCM8-9 for ssDNA-containing DNA structures (Fig. 4a, b).

As an alternative to the MCM2-7 replicative helicase, MCM8-9 has been proposed to support DNA synthesis by driving D-loop extension and migration during gene conversion and break-induced replication^17^. While the biochemical characterization of MCM8-9 has been limited, in vitro helicase activities have been separately reported for MCM8 and MCM9^7,37^. However, their activity as a complex and the identity of factors that may regulate them were previously unknown. In our studies, we found that the helicase activity of the MCM8-9 complex is remarkably stimulated (~6-fold) by the addition of MCM8IP (Fig. 4c, d). This biochemical function of MCM8IP as an activator of MCM8-9 is in line with our and others’ findings that MCM8IP and MCM8-9 physically and genetically interact to promote DNA synthesis and repair upon DNA damage^52^ (Figs. 3f, 5c, 6e, f, and 7d). While the exact mechanism awaits further characterization, the increased ssDNA affinity conferred through MCM8IP interaction may in part underlie the stimulation of MCM8-9 helicase activity, potentially by enhancing processivity. Alternatively, MCM8IP may drive MCM8-9 conformational changes, such as a structural transition from a MCM8-9 heterodimer to a heterohexamer that encircles ssDNA^13,17^. Future studies are required to further define the biochemical properties of the MCM8IP–MCM8-9 complex.

In addition to defining the function of the MCM8IP–MCM8-9 complex in HR, our studies indicate that MCM8IP and its interactions with RPA1 and MCM8-9 are required to maintain replication fork progression in the presence of cisplatin (Fig. 7a–d). Furthermore, MCM8IP promotes the restart of stalled forks upon cisplatin treatment (Supplementary Fig. 9a). The reasons for the impairment in fork progression and restart in the absence of MCM8IP remain to be clarified but possibilities include defective reinitiation of DNA synthesis downstream of DNA crosslinks and impaired replication-coupled crosslink repair.

Upon cisplatin treatment, the MCM8IP–MCM8-9 complex could promote the restart of DNA synthesis by unwinding the parental DNA strands at stalled forks that might have lost the replicative CDC45–MCM2-7–GINS helicase. In line with a possible role for MCM8IP–MCM8-9 as a backup replicative helicase, MCM8IP and MCM8-9 are required for DNA synthesis in the absence of MCM2^17,52^. Fork restart upon cisplatin treatment can involve the repriming of DNA synthesis mediated by the DNA primase-polymerase PRIMPOL^53^. PRIMPOL-mediated repriming of DNA synthesis acts as an alternative pathway to fork reversal^53^, a process that restrains fork progression in the presence of DNA damage to allow sufficient time for the repair of DNA lesions^54^. Further studies are needed to determine whether deficiency in MCM8IP or MCM8-9 may lead to impaired fork repriming and/or accumulation of reversed forks upon cisplatin treatment, thus resulting in fork slowing and arrest.

Cisplatin induces the formation of DNA intra-strand and inter-strand crosslinks (ICLs). ICLs are considered significant barriers to active replication forks that can impede both leading strand synthesis and replicative helicase progression^46,47^. However, a mechanism involving traverse of an ICL by the replisome without significantly compromising fork progression has recently been described^55^. While the exact details of this process need to be elucidated, ICL traverse was shown to depend on CDC45–MCM2-7 complexes^56^. Given the requirement for DNA helicases in ICL traverse, it is possible that MCM8IP–MCM8-9 may promote repriming of DNA synthesis downstream of ICLs as an alternative to CDC45–MCM2-7 complexes.

Besides ICL traverse, ICL repair can also facilitate the restart of DNA synthesis when fork progression is arrested by an ICL^46,47^. ICL repair is mediated by the FA pathway, which coordinates the formation of dual incisions to unhook the crosslink^45,57,58^. Crosslink unhooking generates DSBs that are repaired by HR to restart collapsed forks^59–62^. Previous studies have shown that the localization of MCM8-9 and MCM8IP to cisplatin-induced foci is dependent on the presence of FANCD2^10,52^. These observations raise the possibility that the MCM8IP–MCM8-9 complex may be also required for HR-dependent DNA synthesis during ICL repair.

Cisplatin and other DNA crosslinking agents are frequently utilized for cancer therapy. Our work identifies the importance of the MCM8IP–MCM8-9 complex in mediating cellular resistance to cisplatin and other cancer therapeutic agents, such as olaparib (Fig. 6e, f). Interestingly, MCM8IP and MCM8-9 have recently been identified in genetic screens for mediators of ATR inhibitor and temozolomide resistance^63,64^. Collectively, these studies implicate a broad role for the MCM8IP–MCM8-9 complex in promoting chemoresistance to clinically relevant chemotherapeutic agents. This raises the possibility of targeting MCM8IP or its interaction with MCM8-9 in combination with DNA damaging agents in the treatment of cancer. One possibility is the use of small molecules that inhibit the ATPase activity of MCM8-9 or disrupt the MCM8IP–MCM8-9 interaction. Small peptides that encompass the MCM8-9 binding motif of MCM8IP (Fig. 3e) could also be employed to specifically target the MCM8IP–MCM8-9 interaction.

As previously discussed, *MCM8* and *MCM9* are mutated in POI, an infertility syndrome caused by mutations in at least 60 genes^65^. In line with these findings, *Mcm8-9*-null mice exhibit infertility due to gametogenesis defects^11,12^. Interestingly, *MCM8* and *MCM9* mutations in patient-derived cells cause elevated ICL-induced chromosomal instability^50,51,66^, suggesting that POI may be a consequence of defective MCM8-9-dependent HR events. Our and others’ work implicate the MCM8IP–MCM8-9 complex in HR as well as murine fertility^52^, raising the prospect that mutations in MCM8IP may cause POI. Reciprocally, mutations in MCM8 or MCM9 that disrupt their interaction with MCM8IP may also predispose to POI. Further characterization of the MCM8IP–MCM8-9 complex should reveal important insights into the etiology of POI.

## Methods

### Cell culture and RNAi

HEK293T, HEK293T T-REx, U2OS, U2OS DR-GFP, and U2OS 35S (SCR/RFP) cells were cultured in Dulbecco's modified Eagle's medium supplemented with 10% Fetalgro bovine growth serum (RMBIO). HCT116 cells were cultured in RPMI 1640 supplemented with 10% Fetalgro bovine growth serum. Cells were grown in humidified incubators at 37 °C and 5% CO_2_. CtIP (MQ-011376-00) and nontargeting siRNAs (5′-CCCGCCTGAAGTCTCTGATTAA-3′) were purchased from Dharmacon.

### Plasmids

pDONR223-RPA1 is previously described^24^. MCM8IP cDNA (C17orf53; NM_001171251) amplified from MDA-MB-436 and MCM8 (NM_032485) and MCM9 (NM_017696) cDNAs amplified from HEK293T were recombined into pDONR223 with BP clonase II (Thermo Fisher). Site-directed mutagenesis by inverse polymerase chain reaction was used to generate mutations or introduce epitope tags in MCM8IP and MCM8. All constructs generated for this study were verified by Sanger sequencing. Gateway destination vectors used in this study include pET60-DEST, pMSCV-FLAG-HA-DEST, pHAGE-Ct-FLAG-HA-DEST, and the constructs described below. Gateway recombination was performed with LR Clonase II (Thermo Fisher). Doxycycline-inducible Gateway destination lentiviral vectors for 5′- and 3′-end tagging with BioID were constructed for this study (pHAGE-TREX-NtBioID-DEST and pHAGE-TREX-CtBioID-DEST). Briefly, BioID was amplified from pcDNA3.1 MCS-BirA(R118G)-HA (Addgene #36047) with a 5′- or 3′-end HA-tag and cloned with a linker (13× GGGGS) either upstream or downstream of the attP1/2 recombination cassette of pHAGE-TREX-DEST-puro. For complementation studies in *MCM8IP* KO cells, a Gateway destination lentiviral vector for UbC promoter-driven transgene expression was constructed (pHAGE-UbC-Blast-DEST or pHAGE-UbC-Hygro-DEST). Briefly, the UbC promoter from pPB-UbC^67^ was cloned into the NheI/BamHI sites of pHAGE-TREX-DEST-puro, replacing the CMV promoter and Tet-responsive elements. The puromycin resistance gene was replaced with blasticidin (Bsd) or hygromycin resistance genes by standard cloning techniques. Primers used in this study are listed in Supplementary Data 5.

### Antibodies

The antibodies used in this study for western blotting (WB) and immunofluorescence (IF) are as follows: rabbit anti-C17orf53/MCM8IP (Sigma HPA023393; 1:1000 dilution for WB), mouse anti-C17orf53/MCM8IP (Novus NBP2-37407; 1:500 dilution for WB), rabbit anti-MCM8 (Proteintech 16451-1-AP; 1:5000 dilution for WB), rabbit anti-MCM9 (Millipore ABE2603; 1:10,000 dilution for WB), rabbit anti-RPA1 (Bethyl A300-241A; 1:20,000 dilution for WB), rabbit anti-RPA2 (Bethyl A300-244A; 1:40,000 dilution for WB and 1:10,000 dilution for IF), rabbit anti-SMARCAL1 (Bethyl A301-616A; 1:1000 dilution for WB), mouse anti-SMARCAL1 (Santa Cruz sc-376377; 1:200 dilution for WB), rabbit anti-CtIP (Bethyl A300-488A; 1:1000 dilution for WB), mouse anti-GST (Santa Cruz sc-138; 1:500 dilution for WB), rabbit anti-GST (Abcam ab21070; 1:5000 dilution for WB), mouse anti-FLAG (Sigma F1804; 1:1000 dilution for IF and WB), mouse anti-HA (Sigma H3663; 1:5000 dilution for WB), rat anti-tubulin (Novus NB600-506; 1:20,000 dilution for WB), mouse anti-vinculin (Sigma V9131; 1:10,000 dilution for WB), rabbit anti-Lamin B1 (Thermo Fisher PA5-19468; 1:4000 dilution for WB), rabbit anti-histone H3 (Bethyl A300-823A; 1:2000 dilution for WB), rabbit anti-RAD51 (BioAcademia BAM-70-002; 1:10,000 dilution for IF), mouse anti-cyclin A (Santa Cruz sc-271682; 1:500 dilution for IF), rabbit anti-γH2AX (Bethyl A300-081A; 1:1000 dilution for IF), rat anti-BrdU (Abcam ab6326; 1:100 dilution for IF), and mouse anti-BrdU (BD Biosciences 347580; 1:100 dilution for IF).

### Protein purification

Human MCM8-9, MCM8IP-WT, -MBM #1 and -MBM #2 were expressed in *Spodoptera frugiperda* 9 (*Sf*9) insect cells. MCM9 was cloned into the NotI and SalI sites of pFastBac1-MBP-CtIP-his^68^ to obtain pFastBac1-MBP-MCM9 and MCM8 was cloned into pFastBac1 (Thermo Fisher) using the BamHI and XbaI sites to obtain pFastBac1-FLAG-MCM8. The sequence coding for MCM8 and MCM9 was codon-optimized for expression in *Sf*9 cells (Gen9). Recombinant MCM8-9 was expressed and purified as a complex in *Sf*9 cells by coinfection with baculoviruses prepared from individual pFastBac1 plasmids. MCM8IP WT, MBM #1, and MBM #2 were prepared using the same procedure from pFasBac1 plasmids coding for C-terminal FLAG-tagged proteins. Bacmids, primary and secondary baculoviruses for all constructs were prepared using standard procedures according to manufacturer’s instructions (Bac-to-Bac, Life Technologies). The transfection of *Sf*9 cells was carried out using a Trans-IT insect reagent (Mirus Bio).

For large scale expression and purification of MCM8-9, 800 ml of *Sf*9 cells were seeded at 0.5 × 10^6^ per ml and co-infected with 1:1 ratio of both recombinant baculoviruses. The infected cells were incubated in suspension at 27 °C for 52 h with constant agitation. All purification steps were carried out at 4 °C or on ice. The *Sf*9 cell pellets were resuspended in 3 volumes of lysis buffer containing 50 mM Tris–HCl (pH 7.5), 1 mM dithiothreitol (DTT), 1 mM ethylenediaminetetraacetic acid (EDTA), 1:400 protease inhibitory cocktail (Sigma P8340), phenylmethylsulfonyl fluoride (PMSF) and 30 µg/ml leupeptin for 20 min with continuous stirring. Glycerol was added to 16% (v/v) concentration. Next, 5 M NaCl was added slowly to reach a final concentration of 305 mM. The cell suspension was further incubated for 30 min with continuous stirring, centrifuged at 50,000*g* for 30 min to obtain soluble extract. Pre-equilibrated amylose resin (New England Biolabs) was added to the cleared extract and incubated for 1 h with continuous mixing. The amylose resin was separated from the soluble extract by centrifugation at 2000*g* for 2 min and the supernatant was discarded. The amylose resin was washed extensively batch-wise as well as on disposable columns (Thermo Fisher) with wash buffer (50 mM Tris–HCl, pH 7.5, 2 mM β-mercaptoethanol, 300 mM NaCl, 10% glycerol, 1 mM PMSF). The bound proteins were eluted with elution buffer (50 mM Tris–HCl, pH 7.5, 0.5 mM β-mercaptoethanol, 300 mM NaCl, 10% glycerol, 1 mM PMSF, 10 mM maltose). The eluate was then treated with 1/10 (w/w) PreScission protease for 60 min to cleave off the maltose binding protein (MBP) affinity tag. The cleaved sample was added to pre-equilibrated anti-FLAG M2 Affinity Gel (Sigma A2220) for 1 h with continuous mixing. The FLAG resin was washed extensively on a disposable column (Thermo Fisher) with FLAG wash buffer (50 mM Tris–HCl, pH 7.5, 150 mM NaCl, 10% glycerol, 1 mM PMSF, 0.5 mM β-mercaptoethanol). Finally, recombinant MCM8-9 was eluted from the FLAG resin by FLAG wash buffer supplemented with FLAG peptide (200 ng/µl, Sigma, F4799) and stored at −80 °C.

For large-scale expression and purification of MCM8IP WT and mutants, 1000 ml of *Sf*9 cells were used and the soluble extracts were prepared as described above. Pre-equilibrated anti-FLAG M2 Affinity Gel (Sigma A2220) was added to each of the soluble extract for 1 h with continuous mixing. The FLAG resin was separated and washed batch-wise as described above. The FLAG resin was then washed extensively on a disposable column (Thermo Fisher) with FLAG wash buffer (50 mM Tris–HCl, pH 7.5, 300 mM NaCl, 10% glycerol, 1 mM PMSF, 0.5 mM β-mercaptoethanol, 0.1% NP 40) and then with FLAG wash buffer containing 100 mM NaCl. Finally, the recombinant protein was eluted from the FLAG resin by FLAG wash buffer (with 100 mM NaCl) supplemented with FLAG peptide (200 ng/µl, Sigma F4799) and stored at −80 °C. Human RPA was expressed in *E. coli* and purified using ÄKTA pure (GE Healthcare) with HiTrap Blue HP, HiTrap desalting and HiTrap Q chromatography columns (all GE Healthcare)^69^.

### In vitro interaction studies

Overnight cultures of *E. coli* BL21 (DE3) cells carrying pET60-MCM8IP and its mutants or pET59-MCM8 were diluted into 50 ml of LB to OD_600_ = 0.075 and grown at 30 °C to OD_600_ = 0.5–0.6 (approximately 3 h). Cultures were then induced with isopropyl-β-D-thiogalactoside (IPTG) (1 mM) and grown at 30 °C for an additional 6 h. *E. coli* BL21 (DE3) carrying pCDFDuet-RPA1^24^ were grown overnight at room temperature following induction with IPTG. Cell pellets were lysed in 1 ml wash buffer (20 mM Tris–HCl, pH 7.5, 200 mM NaCl, 0.5% NP40, 1 mM DTT, 10% glycerol) supplemented with a protease inhibitor cocktail (Goldbio GB-330), PMSF (1 mM), lysozyme (0.4 mg/ml), DNase I (20 units, New England Biolabs M0303), and MgCl_2_ (10 mM). Lysates were sonicated (3 ×10 s pulses at 30% output) and then cleared by centrifugation. Cleared lysates from bacteria expressing pET60-MCM8IP and its mutants were incubated with glutathione-agarose beads (Goldbio, G-250) for 2 h at 4 °C with gentle agitation. Immobilized proteins were then washed 4 times and incubated with cleared lysates from bacteria expressing pCDFDuet-RPA1 or pET59-MCM8 for 2 h at 4 °C with gentle agitation. Immobilized protein-complexes were then washed 5 times, eluted with LDS sample buffer, and resolved by sodium dodecyl sulfate polyacrylamide gel electrophoresis (SDS-PAGE).

To study the interaction between recombinant MCM8-9 and MCM8IP, *Sf*9 cells were infected with MBP-MCM9 and FLAG-MCM8 baculoviruses (see “Protein purification”). Cells were lysed and MCM8-9 was immobilized on amylose resin (New England Biolabs) and washed with wash buffer (50 mM Tris–HCl, pH 7.5, 2 mM β-mercaptoethanol, 300 mM NaCl, 0.1% (v/v) NP40, 1 mM PMSF). Resin-bound MCM8-9 was then incubated with 1 µg of MCM8IP, either wild-type or MBM #1 mutant, diluted in binding buffer (50 mM Tris–HCl, pH 7.5, 2 mM β-mercaptoethanol, 3 mM EDTA, 100 mM NaCl, 0.2 µg/µl bovine serum albumin (BSA), 1 mM PMSF) for 1 h at 4 °C with continuous rotation. The resin was washed 4 times with wash buffer containing 100 mM NaCl, proteins were eluted in wash buffer supplemented with 10 mM maltose and detected by western blotting. As a negative control, MCM8IP was incubated with the resin without the bait protein.

### Immunoprecipitation

Immunoprecipitation was performed as reported^24^. Briefly, HEK293T cells transduced with retroviruses carrying pMSCV-FLAG-HA-RPA1, -MCM8IP, -MCM8, -MCM9, or a -GFP control were grown to near confluency in a 10 cm dish and harvested in phosphate-buffered saline (PBS). Cell pellets were resuspended in 750 µl of mammalian cell lysis buffer (MCLB) (50 mM Tris–HCl, pH 7.5, 1% NP40) supplemented with 150 mM NaCl, and protease and phosphatase inhibitor cocktails (Goldbio, GB-331 and GB-450). Following incubation for 30 min at 4 °C with gentle agitation, cell lysates were cleared by centrifugation and the low-salt supernatant collected. Cell pellets were then resuspended in 250 µl of MCLB supplemented with 500 mM NaCl and protease and phosphatase inhibitors and gently agitated for 1 h at 4 °C. After centrifugation, the salt concentration of the supernatant was adjusted to 150 mM NaCl and combined with the low-salt supernatant. The combined lysates were then incubated with 20 µl of anti-HA agarose beads (Sigma A2095) for 4 h at 4 °C with gentle agitation. Protein-bound beads were subsequently washed 4 times in buffer (50 mM Tris–HCl, pH 7.5, 1% NP-40 and 150 mM NaCl) and bound proteins eluted in LDS sample buffer. For mass spectrometry, HEK293T cells stably transduced with pHAGE-MCM8IP-FLAG-HA or pHAGE-GFP-FLAG-HA were grown to near confluency in two 15 cm dishes and the method described above was scaled up accordingly.

### Proximity-dependent labeling with BioID

Small-scale BioID experiments were performed with cells grown to near confluency in 10 cm dishes. Briefly, HEK293T T-REx cells transduced with lentivirus carrying pHAGE-TREX-BioID control or pHAGE-TREX-BioID-RPA1 were treated with 1 µg/ml doxycycline for 24 h. Cells were then treated with DNA damaging agents or left untreated in media supplemented with 50 µM biotin and 1 µg/ml doxycycline for an additional 18–24 h. Cells were washed 3 times and harvested in PBS. Cell pellets were resuspended in 1 ml of radioimmunoprecipitation assay (RIPA) buffer (50 mM Tris–HCl, pH 7.5, 150 mM NaCl, 1% NP40, 0.5% deoxycholic acid, 0.1% SDS, 1 mM EDTA, 1 mM DTT) supplemented with protease and phosphatase inhibitors. Lysates were then sonicated (3 ×10 s pulses at 30% output), and treated with benzonase for 30 min at 4 °C with gentle agitation. Following centrifugation, cleared lysates were incubated with 20 µl of streptavidin-coated magnetic beads (Thermo Fisher 65001) for 4 h at 4 °C with gentle agitation. Protein-bound beads were separated using a magnetic rack, washed 4 times with RIPA buffer, and eluted with LDS sample buffer supplemented with biotin.

To identify RPA1 interactors by mass spectrometry, HEK293T T-REx cells expressing pHAGE-TREX-BioID-RPA1 or the BioID-alone control were grown to near confluency in three 15 cm dishes and labeled and harvested as described above. Cells from each sample were then evenly distributed among seven microfuge tubes. Each cell pellet was resuspended in 1 ml of lysis buffer (6 M Urea, 50 mM Tris–HCl, pH 7.5, 0.5% Triton X-100) supplemented with 1 mM DTT and protease inhibitors, sonicated (3 ×10 s pulses at 30% output) and cleared by centrifugation. Lysates were then incubated with 40 µl of streptavidin-coated magnetic beads per tube (280 µl total beads per sample) overnight at 4 °C with gentle agitation. The next day, protein-bound beads for each sample were pooled and washed 4 times with lysis buffer and another 4 times with lysis buffer lacking Triton X-100 while exchanging fresh microfuge tubes with each wash. All remaining buffer from the final wash was removed by aspiration.

To identify MCM8IP interactors by mass spectrometry, HEK293T T-REx cells expressing pHAGE-TREX-BioID-MCM8IP, pHAGE-TREX-MCM8IP-BioID, or the BioID-alone control were grown to near confluency in three 15 cm dishes and labeled and harvested as above. Following the even distribution of cells into seven microfuge tubes, each cell pellet was resuspended in 1 ml RIPA buffer and lysates were prepared as described above for small-scale purification. Prepared lysates were incubated with 20 µl of streptavidin beads per tube (140 µl total beads per sample) for 4 h at 4 °C with gentle agitation. Protein-bound beads were pooled and subsequently washed 4 times with RIPA buffer and another 4 times with detergent-free buffer (50 mM Tris–HCl, pH 7.5, 150 mM NaCl) while exchanging fresh microfuge tubes with each wash. All remaining buffer from the final wash was removed by aspiration.

### Sample preparation for mass spectrometry

Proteins bound to streptavidin beads were washed 5 times with 200 µl of 50 mM ammonium bicarbonate and subjected to disulfide bond reduction with 5 mM DTT (56 °C, 30 min) and alkylation with 10 mM iodoacetamide (IAA) (room temperature, 30 min in the dark). Excess IAA was quenched with 5 mM DTT (room temperature, 15 min in the dark). Proteins bound on beads were digested overnight at 37 °C with 1 µg of trypsin/LysC mix. The next day, digested peptides were collected in a new microfuge tube, digestion was stopped by the addition of 1% trifluoroacetic acid  (final v/v), and samples were then centrifuged at 14,000*g* for 10 min at room temperature. Cleared digested peptides were desalted on a SDB-RP StageTip and dried in a speed-vac. Dried peptides were dissolved in 3% acetonitrile/0.1% formic acid.

Immunoprecipitated samples were separated on 4–12% gradient SDS-PAGE and stained with SimplyBlue (Thermo Fisher). Protein gel slices were excised and in-gel digestion performed. Gel slices were washed with 1:1 (acetonitrile:100 mM ammonium bicarbonate) for 30 min and then dehydrated with 100% acetonitrile for 10 min until shrinkage. Excess acetonitrile was removed and slices were dried in a speed-vac for 10 min without heat. Gel slices were then reduced with 5 mM DTT for 30 min at 56 °C in an air thermostat and chilled down to room temperature before alkylation with 11 mM IAA for 30 min in the dark. Gel slices were washed with 100 mM ammonium bicarbonate and 100% acetonitrile for 10 min each. Excess acetonitrile was removed and dried in a speed-vac for 10 min without heat. Gel slices were then rehydrated in a solution of 25 ng/µl trypsin in 50 mM ammonium bicarbonate on ice for 30 min. Digestions were performed overnight at 37 °C in an air thermostat. Digested peptides were collected and further extracted from gel slices in extraction buffer (1:2 v/v, 5% formic acid/acetonitrile) with high-speed shaking in an air thermostat. Supernatant from both extractions was combined and dried down in a speed-vac. Dried peptides were dissolved in 3% acetonitrile/0.1% formic acid.

### LC–MS/MS analysis

Thermo Scientific UltiMate 3000 RSLCnano system, Thermo Scientific EASY Spray source with Thermo Scientific Acclaim PepMap100 2 cm × 75 µm trap column, and Thermo Scientific EASY-Spray PepMap RSLC C18 were used for peptide preparation. 50 cm ×75 µm ID column were used to separate desalted peptides with a 5–30% acetonitrile gradient in 0.1% formic acid over 50 min or 127 min at a flow rate of 250 nl/min. After each gradient, the column was washed with 90% buffer B (0.1% formic acid, 100% HPLC-grade acetonitrile) for 5 min and re-equilibrated with 98% buffer A (0.1% formic acid, 100% HPLC-grade water) for 40 min.

Thermo Scientific Q Exactive HF mass spectrometer was used for peptide MS/MS analysis of BirA*–RPA1 and BirA* control. MS data were acquired with an automatic switch between a full scan and 15 data-dependent MS/MS scans (TopN method). Target value for the full scan MS spectra was 3 × 10^6^ ions in the 375–2000 *m*/*z* range with a maximum injection time of 100 ms and resolution of 60,000 at 200 *m*/*z* with data collected in profile mode. Precursors were selected using a 1.6 *m*/*z* isolation width. Precursors were fragmented by higher-energy C-trap dissociation with normalized collision energy of 27 eV. MS/MS scans were acquired at a resolution of 15,000 at 200 *m*/*z* with an ion target value of 2 × 10^5^, maximum injection time of 50 ms, dynamic exclusion for 15 s and data collected in centroid mode.

Thermo Scientific Orbitrap Fusion Tribrid mass spectrometer was used for peptide MS/MS analysis of BirA*–MCM8IP, MCM8IP–BirA* and MCM8IP-HA and respective controls. Survey scans of peptide precursors were performed from 400 to 1500 *m*/*z* at 120 K FWHM resolution (at 200 *m*/*z*) with a 4 × 10^5^ ion count target and a maximum injection time of 50 ms. The instrument was set to run in top speed mode with 3 s cycles for the survey and the MS/MS scans. After a survey scan, tandem MS was performed on the most abundant precursors exhibiting a charge state from 2 to 6 of greater than 5 × 10^3^ intensity by isolating them in the quadrupole at 1.6 Th. CID fragmentation was applied with 35% collision energy and resulting fragments were detected using the rapid scan rate in the ion trap. The AGC target for MS/MS was set to 1 × 10^4^ and the maximum injection time limited to 35 ms. The dynamic exclusion was set to 45 s with a 10 ppm mass tolerance around the precursor and its isotopes. Monoisotopic precursor selection was enabled. Each sample was analyzed twice by mass spectrometry (technical duplicate).

### Mass spectrometry data analysis

Raw mass spectrometric data were analyzed using MaxQuant^70^ v.1.6.1.0 and Andromeda^71^ employed for database search at default settings with a few modifications. The default was used for first search tolerance and main search tolerance: 20 and 6 ppm, respectively. MaxQuant was set up to search the reference human proteome database downloaded from UniProt. MaxQuant performed the search for trypsin digestion with up to two missed cleavages. Peptide, Site, and Protein false discovery rates (FDR) were all set to 1% with a minimum of two peptides needed for identification but two peptides needed to calculate a protein level ratio. Carbamidomethyl modification of cysteine was used as a fixed modification, while oxidation of methionine (M), deamination of asparagine or glutamine (NQ), and acetylation on N-termini of proteins were used as variable modifications. MaxQuant combined folders were uploaded into Scaffold 4 for data visualization. Spectral counting was used for analysis to compare samples. Naturally biotinylated carboxylases were specifically excluded from the analysis of BioID experiments^20^. From Scaffold, identified proteins (1% FDR, minimum of two unique peptides) were considered putative RPA interactors if total spectral counts were at least 3-fold enriched in BirA*–RPA1 relative to BirA* alone. For the identification of putative MCM8IP interactors, identified proteins (1% FDR, minimum of two unique peptides in each of two technical replicates) were considered hits if the average total spectral count of two technical replicates was at least 5-fold enriched in BirA*–MCM8IP, MCM8IP–BirA*, or MCM8IP-HA relative to their respective controls (BirA* alone or GFP-HA). Total numbers of putative interactors stated in this study for RPA1 (305) and MCM8IP (Fig. 3a) reflect unique protein identifications where multiple isoforms of proteins found to be enriched were counted as a single hit. However, all enriched protein isoforms are listed in Supplementary Data 1 and 2.

### DNA substrate preparation

For the DNA binding experiments, single-stranded DNA oligonucleotide (93 nt long, X12-3HJ3)^69^ was labeled at the 5′ terminus with [γ-^32^P] ATP and T4 polynucleotide kinase (New England Biolabs), according to standard protocols. Unincorporated nucleotides were removed using Micro Bio-Spin™ P-30 Gel Columns (Bio-Rad). Plasmid length DNA binding experiments were performed with unlabeled M13mp18 single-stranded DNA (New England Biolabs).

For helicase assays, oligonucleotide containing a 37 nt region complementary to the M13mp18(+) strand (nucleotides 6289–6326) and a 40 nt tail at the 5′ end was annealed to M13mp18 single-stranded DNA to prepare the substrate^38^. The oligonucleotide was labeled at the 3′ terminus [α-^32^P] dCTP (Perkin Elmer) and terminal transferase (New England Biolabs) before annealing according to the standard procedures.

### Electrophoretic mobility shift assays

Binding reactions (15 µl volume) were carried out in 25 mM Tris acetate, pH 7.5, 3 mM EDTA, 1 mM DTT, 100 µg/ml BSA (New England Biolabs), single-stranded oligonucleotide DNA substrate (1 nM, molecules). Proteins were added and incubated for 15 min on ice. Loading dye (5 µl; 50% glycerol, bromophenol blue) was added to reactions and products were separated on 4% polyacrylamide gels (ratio acrylamide:bisacrylamide 19:1, Bio-Rad) in TAE buffer at 4 °C. The gels were dried on 17 CHR (Whatman), exposed to a storage phosphor screen (GE Healthcare) and scanned by a Typhoon Phosphor Imager (FLA9500, GE Healthcare). DNA binding experiments using plasmids were performed under the same conditions except with 100 ng DNA per reaction and were run on 0.8% agarose gel at 4 °C and post-stained with GelRed (Biotium).

### Helicase assays

Helicase assays (15 µl volume) were performed in a reaction buffer (25 mM HEPES, pH 8.5, 25 mM sodium acetate, pH 5.2, 1 mM magnesium acetate, 4 mM ATP, 1 mM DTT, 0.1 mg/ml BSA) with 0.1 nM (in molecules) DNA substrate. Recombinant proteins were added as indicated. RPA was added after incubating the DNA and all other proteins for 15 min at 37 °C. The reactions were further incubated at 37 °C for 45 min and stopped using 2% stop buffer containing (2% SDS, 150 mM EDTA, and 30% glycerol) and 1 µl of proteinase K (14–22 mg/ml, Roche) by incubating at 37 °C for 10 min. To avoid re-annealing of the substrate, the stop solution was supplemented with a 200-fold excess of the unlabeled oligonucleotide with the same sequence as the ^32^P-labeled one. The products were separated by 10% polyacrylamide gel electrophoresis in TBE buffer, dried on 17 CHR chromatography paper (Whatman) and analyzed as described above.

### Immunofluorescence

For UV laser microirradiation experiments, U2OS cells expressing pMSCV-FLAG-HA-MCM8IP or pHAGE-Ct-FLAG-HA-MCM8IP and mutants were seeded at a density of 30,000 cells per well in 8-well chamber slides (Nunc). Cells were pre-sensitized with 10 µM BrdU for at least 24 h. UV laser microirradiation was performed using an Inverted Zeiss AxioObserver.Z1 with PALM MicroBeam IV (cutting parameters: focus 65%, energy 47%, Speed 77%). Following microirradiation, cells were incubated for 2–3 h at 37 °C and then simultaneously fixed and permeabilized (2% formaldehyde, 0.5% Triton X-100 in PBS) for 25 min. Cells were incubated with anti-FLAG and anti-γH2AX overnight in blocking buffer (3% BSA, 0.05% Triton-X100 in PBS). Cells were then stained with appropriate secondary antibodies conjugated with Alexa 488 or Alexa 594 and mounted with Fluoroshield with DAPI. For cisplatin-induced MCM8IP foci formation experiments, HCT116 *MCM8IP* KO cells expressing pHAGE-UbC-Hygro-MCM8IP WT-3xFLAG and mutants were initially seeded at sub-confluency on glass coverslips, then treated with 10 µM cisplatin or vehicle for 24 h the following day, and subsequently fixed and stained as above. Cells were imaged with a Nikon Eclipse 90i microscope equipped with an industrial camera (The Imaging Source DMK 33UX174) and operated with Nikon NIS Elements software. Images were analyzed in ImageJ.

For RAD51 and RPA immunofluorescence studies, HCT116 cells were seeded on black 96-well bottom-glass plates and the day after treated or not with 10 µM cisplatin or subjected to ionizing radiation (8 Gy). Six hours after irradiation or 24 h after cisplatin treatment, cells were simultaneously fixed and permeabilized (4% paraformaldehyde, 0.5% Triton X-100) for 10 min at room temperature. Cells were incubated in blocking solution (3% BSA in TBS-Tween 0.1%) for 1 h and then in primary antibody diluted in blocking solution for 1 h at room temperature or overnight at 4 °C. Primary anti-RAD51 or anti-RPA2 and anti-cyclin A were used for staining. Cells were washed 3 times with TBS-T and then incubated for 1 h at room temperature with the appropriate secondary antibody, Alexa Fluor 488-labeled anti-rabbit and Alexa Fluor 594-labeled goat anti-mouse at 1:1000 dilution (Thermo Fisher A-11008 and A-11005). After three washes in TBS-T, cells were incubated with DAPI for 5 min at room temperature to counterstain nuclei. Two-dimensional acquisitions were made using the ImageXpress Nano Automated Imaging System microscope (Molecular Devices) equipped with a 40× Plan Apo objective (0.95 numerical aperture). An integrated imaging software (MetaXpress 6) was used for image analysis. The total number of RAD51 or RPA foci per cell was measured in cyclin A-positive and -negative cells. At least 1000 cells per experimental point were counted, and each experiment was repeated at least 2 times independently.

### Subcellular fractionation

Subcellular fractionation was performed as described^72^ with some modifications. Briefly, cells were harvested by trypsinization, washed with PBS, and resuspended in CSK buffer (10 mM PIPES, pH 6.8, 100 mM NaCl, 1 mM EGTA, 1 mM EDTA, 300 mM sucrose, 1.5 mM MgCl_2_, 0.1% Triton X-100, 1 mM DTT) supplemented with protease and phosphatase inhibitors. After 5 min of incubation on ice, soluble and insoluble fractions were separated by centrifugation (1500*g*, 5 min, 4 °C). The supernatant (soluble fraction) was collected and the pellet was washed once with CSK buffer. After centrifugation, the supernatant was removed and the pellet (chromatin fraction) was resuspended in LDS sample buffer.

### CRISPR-Cas9 gene targeting

Guide RNAs targeting MCM8IP, MCM8, and MCM9 were designed using GPP sgRNA Designer (Broad Institute). The targeted sequences are as follows: MCM8IP #1 (5′-CGACCCCCCTTGAGACCTGGT-3′), MCM8IP #2 (5′-TTCAGTATTGGCTAAAAAAGC-3′), MCM8IP #3 (5′-CAGCTGGATTGGCAATCAGAG-3′), MCM8 #1 (5′-CACGTGGCGTGTATGTTTGT-3′), MCM8 #2 (5′-GTGTGTCGAGGCAGGTCATT-3′), MCM9 #1 (5′-ACGGGATTGTAATGCAACGG-3′), and MCM9 #2 (5′-ACACTGTCTGATGTGGGCAA-3′). MCM8IP sgRNAs were cloned into the BsmBI/Esp3I sites of pXPR206 (Addgene #96920). MCM8 and MCM9 sgRNAs were cloned into the BsmBI/Esp3I sites of pLentiCRISPR v2 Blast (Addgene #98293). Following stable lentiviral transduction of cells, targeting efficiencies were evaluated by western blotting.

### Homologous recombination assays

U2OS DR-GFP^40^ or U2OS 35S (SCR/RFP)^44^ cells (gift from Ralph Scully) were seeded at a density of 250,000–300,000 cells per well in 6-well plates. The following day, cells were transfected (Mirus LT-1) with 2.5 µg of an *I-SceI*-expression vector or an empty vector control (gifts from Shan Zha). Parallel transfections with pEGFP-N3 (gift from Jean Gautier) were performed to assess transfection efficiency. Two days after transfection, cells were harvested by trypsinization and resuspended in PBS in preparation for flow cytometry. Approximately 20,000 DR-GFP cells and 200,000 SCR/RFP cells were analyzed per sample on a BD LSRII or BD LSRFortessa cytometer, respectively. GFP and/or RFP-positive populations determined by flow cytometry were normalized for transfection efficiency. Gene conversion events are presented as repair efficiencies relative to the *I-SceI*-transfected non-targeting control or WT cDNA-complemented *MCM8IP* KO cells.

For experiments using the BFP reporter, BFP-positive HEK293T cells were seeded at 50–70% confluency into 24-well plates and transfected by mixing TransIT-293T (3 µl; Mirus) and 250 ng plasmid pX330 containing a BFP targeting sgRNA along with a plasmid HDR donor (500 ng). The cells were collected 3 days after transfection and analyzed by flow cytometry for GFP-positive cells on a BD LSRFortessa.

### Survival assays

Survival assays were performed as reported^73^. Briefly, HCT116 cells were seeded at a density of 5000 cells per well in 12-well plates. The next day, cells were treated with DNA damaging agents and allowed to grow for an additional 5–7 days. Following fixation (10% methanol and 10% acetic acid in water) and staining with crystal violet (1% w/v in methanol), plates were washed thoroughly and allowed to fully dry. Cells were subsequently destained (0.1% w/v SDS in methanol) and the resuspended solution transferred to a 96-well plate for quantification with a spectrophotometer (*λ* = 595 nm). Following background subtraction, cell survival is presented as a percentage of the untreated control.

### DNA fiber analysis

To measure fork elongation rate, exponentially growing HCT116 cells were pulse-labeled with 25 µM CldU (5 min), washed in warm 1× PBS and exposed to 125 µM IdU with or without 30 µM cisplatin (40 min). Alternatively, to measure fork stalling, cells were pulse-labeled with 25 µM CldU (5 min), exposed to 30 µM cisplatin for 1 h and 35 min in the presence of CldU, washed in warm 1× PBS and exposed to 125 µM IdU (40 min). Labeled cells were trypsinized and resuspended in ice-cold PBS at 2 × 10^5^ cells/ml. Two microliters of this suspension were spotted onto a pre-cleaned glass slide and lysed with 10 µl of spreading buffer (0.5% SDS in 200 mM Tris–HCl, pH 7.4 and 50 mM EDTA). After 6 min, the slides were tilted at 15° relative to horizontal, allowing the DNA to spread. Slides were air-dried, fixed in methanol and acetic acid (3:1) for 2 min, rehydrated in PBS for 10 min and denatured with 2.5 M HCl for 1 h at room temperature. Slides were then rinsed in PBS and blocked in PBS + 0.1% Triton X-100 (PBS-T) + 3% BSA for 1 h at room temperature. Rat anti-BrdU and mouse anti-BrdU were then applied to detect CldU and IdU, respectively. After a 2-h incubation, slides were washed in PBS and stained with Alexa Fluor 488-labeled goat anti-mouse IgG1 antibody and Alexa Fluor 594-labeled goat anti-rat antibody (1:300 each, Thermo Fisher). Slides were mounted in Prolong Gold Antifade (Thermo Fisher) and stored at −20 °C. Replication tracks were imaged on a Nikon Eclipse 90i microscope fitted with a PL Apo 40×/0.95 numerical aperture (NA) objective and measured using ImageJ software. In each experiment, 100 or more individual tracks were measured for fork elongation rate estimation, more than 400 individual tracks were analyzed for fork stalling estimation. Each experiment was repeated at least 2 times independently.

### Phylogenetic analyses

We located *MCM8IP* gene orthologs in distant species using BLASTp, and in difficult cases PSI-BLAST, starting with either the full protein sequence from human, or a truncated human conserved domain spanning amino acid positions 381–575. We targeted species’ genomes that were assembled to high quality within the major branches of the eukaryote tree of life. We searched all major opisthokont lineages that had such high-quality nuclear genome sequences and also included the closely related ameobozoa and green plant groups. When no sequence was found in a high-quality genome sequence, the entire taxonomic group was also searched via Taxonomic group filtering available on the BLAST hosted at NCBI. A full list of species is found in Supplementary Data 3. Attempts to find *MCM8IP* in more distant taxonomic groups were not successful.

To test for correlated gene loss, we compared two nested likelihood models of binary trait evolution (independent and dependent) implemented in *BayesTraits*^74,75^. The independent model had two free parameters to estimate, the loss rate of gene 1 and the loss rate of gene 2. We set the parameters for gain of traits (alphas) to zero with the Restrict alpha1 alpha2 0 command, since it is not possible to gain one of these genes given how we define the *MCM8*, *MCM9* and *MCM8IP* genes as being inherited strictly as orthologs. The dependent model then divides the two loss rate parameters into loss in the presence or absence of the other gene, resulting in four free parameters. Rates corresponding to gene gain were set to zero with the Restrict q12 q13 q24 q34 0 command.

Each model was provided with a phylogenetic tree reflecting the speciation patterns leading to the studied species. The tree was downloaded from TimeTree and matches the major relationships published for these groups^76,77^. Gene presence/absence data were provided to the models for each pair of genes separately and the models were optimized with 100,000 maximum likelihood tries to ensure model convergence. Maximum likelihood values of the two nested models were compared with a likelihood ratio test and the *p*-value was estimated with the chi-square distribution with 2 degrees of freedom. A low *p*-value was taken as evidence of rejection of the independent model in favor of the dependent model for that gene pair.

### Coevolutionary analyses

Evolutionary rate covariation (ERC) values were calculated for protein pairs as the correlation coefficient of branch-specific evolutionary rates using protein sequences from 33 mammalian species^78^. ERC between MCM8IP and major mammalian DNA repair pathways was calculated by taking the mean ERC score between MCM8IP and the genes in each pathway. Statistical significance was determined by permutation test, where the *p*-value is the computed probability of the observed mean ERC or greater when taking the mean between each gene group and 10,000 random genes, as described^79^.

### Statistics and reproducibility

Primary data were recorded using Microsoft Excel and statistical analyses performed using GraphPad Prism 6. Exact *p*-values are indicated in the Source Data file. Results presented without statistical analyses were repeated as follows: Figs. 1c–f, 2d, e, 3a, f, and 6b, g, h and Supplementary Figs. 1d, e, 4e, 5f, g, and 6b, d, e are representative of three or more independent experiments. Figures 2a, f, 3d, and 7f and Supplementary Figs. 1b, c, 2b, 3a–c, 7c, f, 8a, and 9c are representative of two independent experiments, while results in Supplementary Figs. 1a and 5a–c were conducted once.

### Reporting summary

Further information on research design is available in the Nature Research Reporting Summary linked to this article.

## Supplementary information

Supplementary Information

Description of Additional Supplementary Files

Supplementary Data 1

Supplementary Data 2

Supplementary Data 3

Supplementary Data 4

Supplementary Data 5

Reporting Summary

## Data Availability

The mass spectrometry data obtained in this study are available in PRIDE (PXD019156 [https://www.ebi.ac.uk/pride/]). The source data underlying Figs. 1c–e, g, 2a, c, d, f, 3b, d, f, 4a–f, 5b–d, f, 6a–i, and 7b, d, f and Supplementary Figs. 1b–d, 2b, 3a–c, 4b, d, e, 5a–g, 6a, b, d, e, 7b, c, e, f, 8a–e, and 9a, b are provided as a Source Data file. All unique reagents and data generated in this study will be made available from the authors upon request. Source data are provided with this paper.

## Source data

Source Data
