# Supplementary material for: MCM8IP activates the MCM8-9 helicase to promote DNA synthesis and homologous recombination upon DNA damage
Source: Nat Commun. 2020 Jun 11;11:2948. doi: 10.1038/s41467-020-16718-3 (PMC7290032; doi:10.1038/s41467-020-16718-3)
Supplement: Supplementary file 1 — Supplementary Information [file 41467_2020_16718_MOESM1_ESM.pdf]

SUPPLEMENTARY INFORMATION

MCM8IP activates the MCM8-9 helicase to promote DNA synthesis and homologous recombination upon DNA damage

Huang et al.

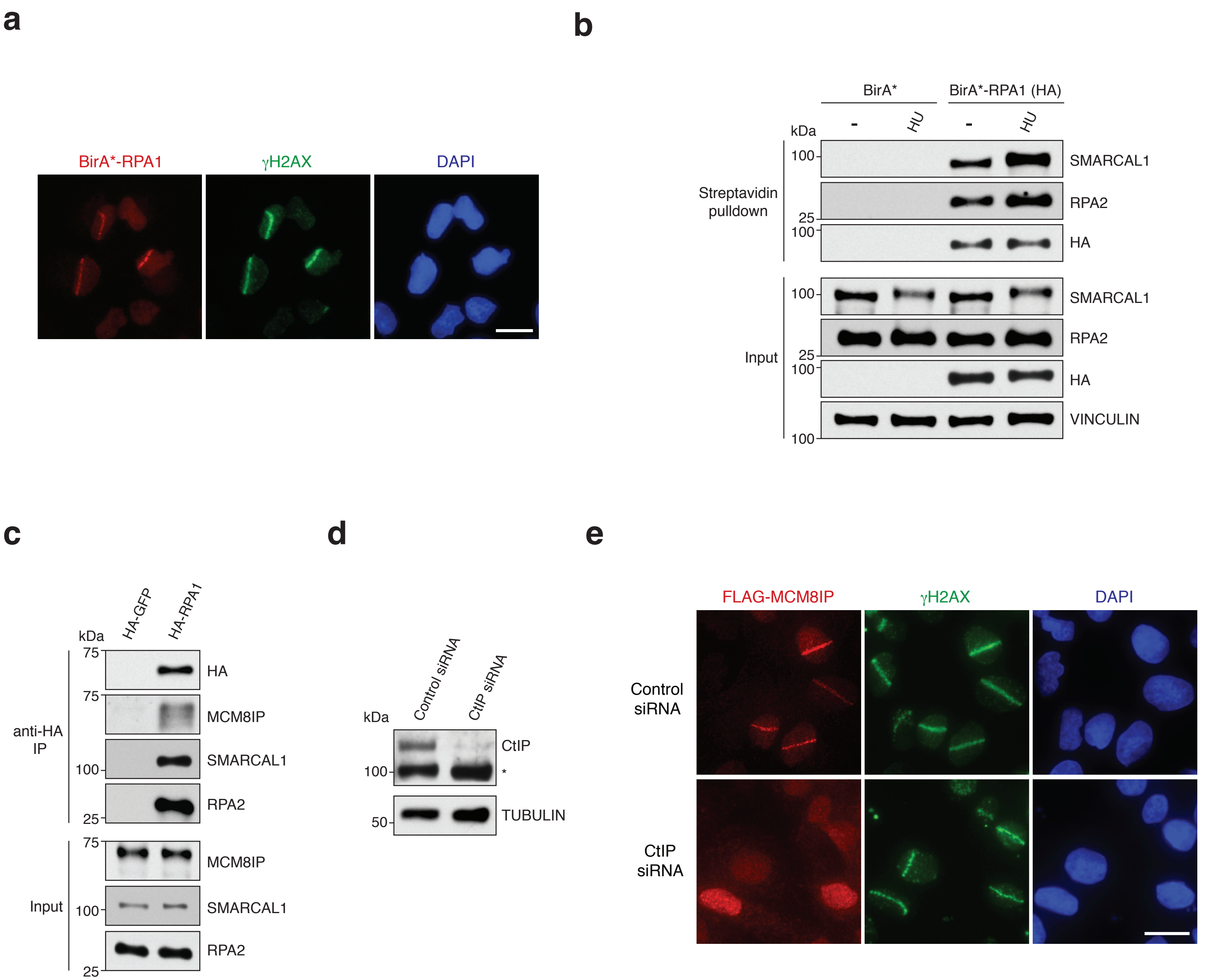

Supplementary Figure 1

Supplementary Figure 1. Interaction and localization studies for RPA1 and MCM8IP

a Representative images of HA-BirA*-RPA1 recruitment to sites of DNA damage 1 hour after UV laser microirradiation in U2OS cells. DNA damage tracts are indicated with γH2AX staining. Scale bar = 20 μm.

b Detection by western blot of RPA2 and SMARCAL1 in streptavidin pulldowns from HEK293T T-REx cells expressing doxycycline-inducible BirA* or BirA*-RPA1. Cells were treated with HU (1 mM) in the presence of exogenous biotin for 24 hours prior to lysis. Vinculin is shown as a loading control.

c Detection by western blot of MCM8IP, SMARCAL1 and RPA2 co-immunoprecipitated by HA-GFP or HA-RPA1 from HEK293T cells.

d Detection by western blot of CtIP in U2OS cells expressing FLAG-MCM8IP transfected with control or CtIP siRNA and subjected to UV laser microirradiation, as shown in **e**. Tubulin is shown as a loading control. Asterisk indicates a non-specific band.

e Representative images of FLAG-MCM8IP recruitment to sites of UV laser microirradiation in U2OS cells transfected with control or CtIP siRNA, as quantified in Figure **1g**. DNA damage tracts are indicated with γH2AX staining. Scale bar = 20 μm.

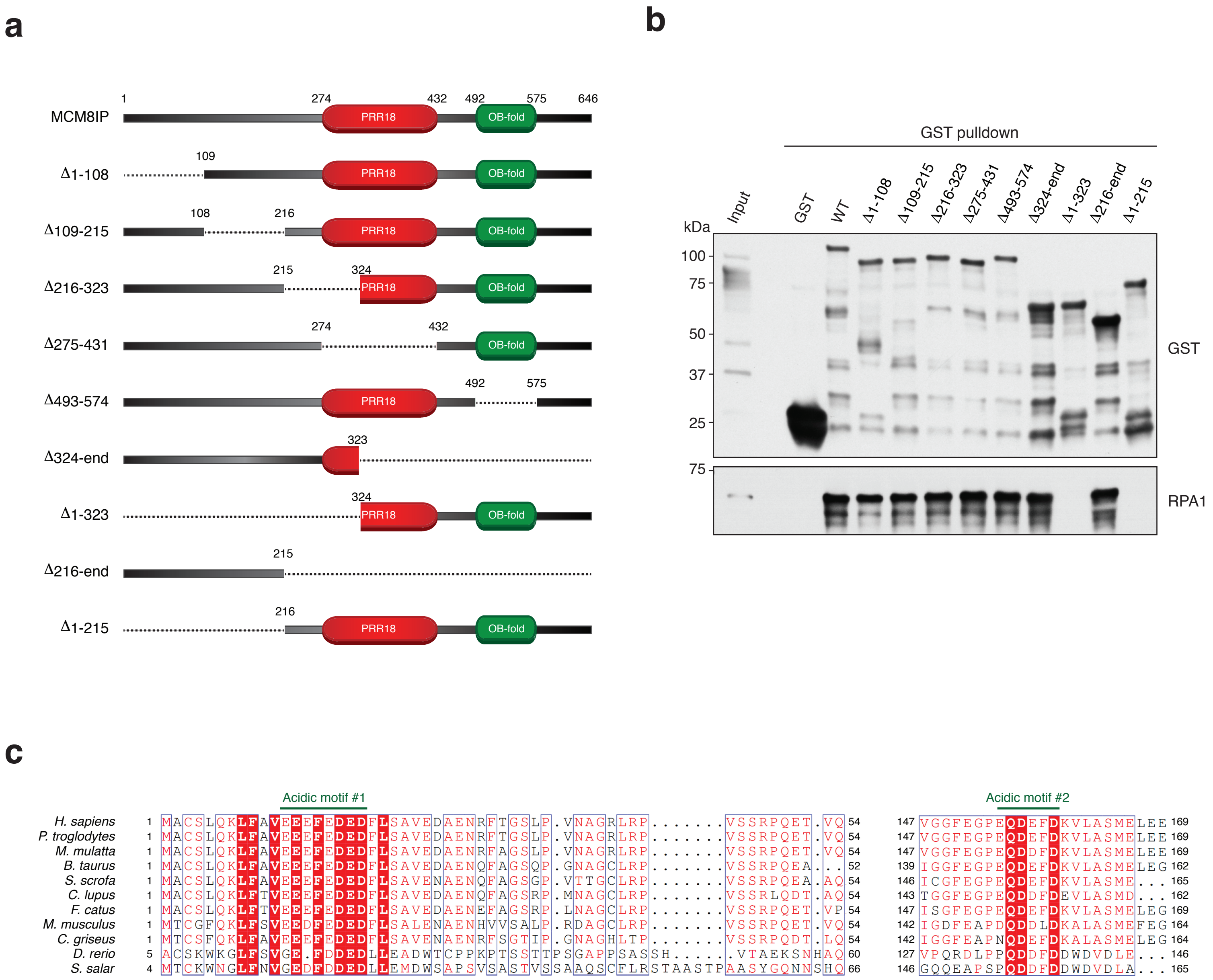

Supplementary Figure 2

Supplementary Figure 2. Identification of RPA1-binding motifs in MCM8IP

a Schematic representation of full-length MCM8IP and MCM8IP deletion mutants. Shown in red and green are the PRR18 and DUF4539 (a predicted OB-fold) domains, respectively.

b Detection by western blot of RPA1 co-precipitated by bead-bound recombinant GST, GST-MCM8IP WT or GST fused to MCM8IP mutants presented in **a**.

c Alignment from various species of two conserved acidic motifs within the first 215 amino acids of human MCM8IP predicted to interact with RPA1. Sequence alignments were conducted using Clustal Omega and processed using ESPript.

a

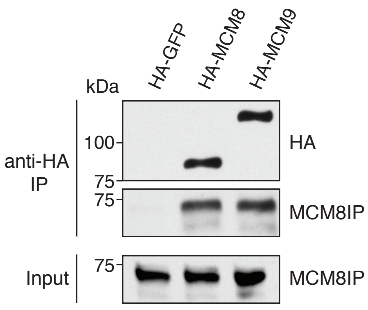

b

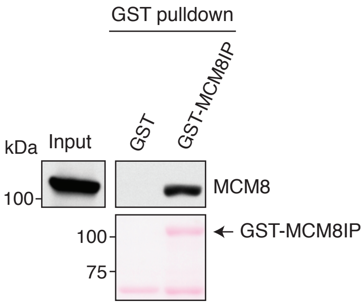

c

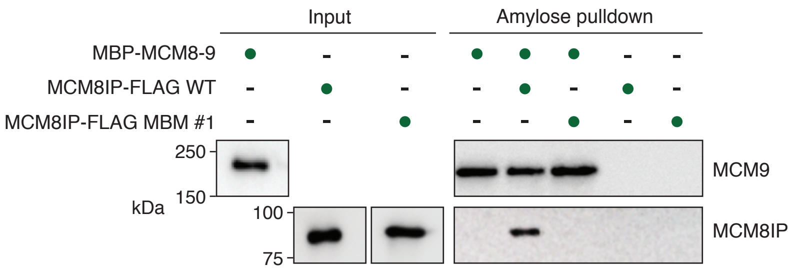

d

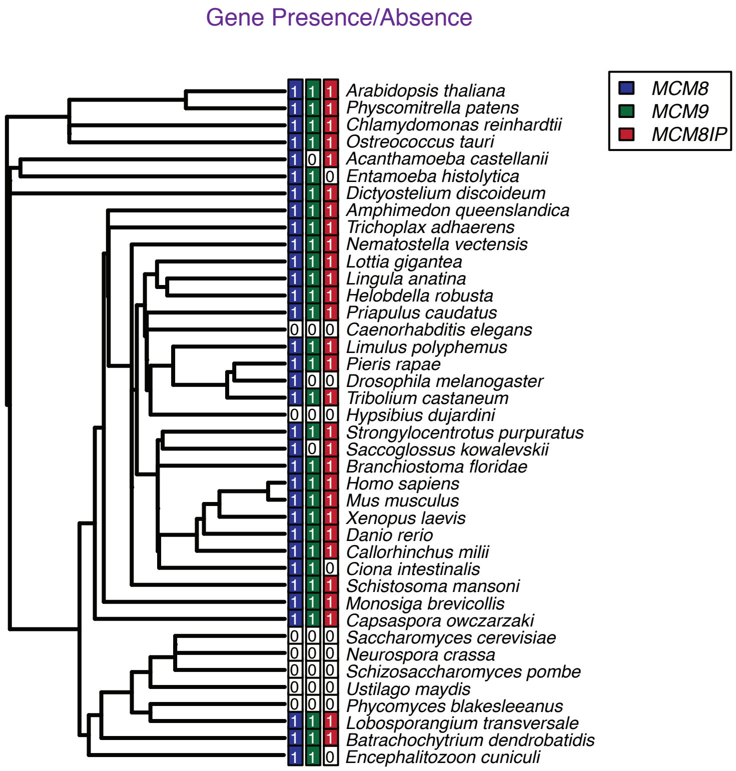

Supplementary Figure 3

Supplementary Figure 3. Characterization of the MCM8IP-MCM8-9 interaction

a Detection by western blot of MCM8IP co-immunoprecipitated with HA-GFP, HA-MCM8 or HA-MCM9 from HEK293T cells.

b Detection by western blot of recombinant HIS-MCM8 co-precipitated by bead-bound recombinant GST or GST-MCM8IP.

c Detection by western blot of recombinant MCM8IP-FLAG, either WT or MBM #1 mutant, co-precipitated by amylose bead-bound recombinant MBP-MCM8-9 complex.

d Phylogenetic analysis indicating the presence or absence of *MCM8IP*, *MCM8* and *MCM9* in various species. This species phylogeny represents most major taxonomic groups of opisthokonts, amoebas, and green plants. Color-filled squares containing “1” indicate the presence of the gene in the indicated species. See also Supplementary Data 3.

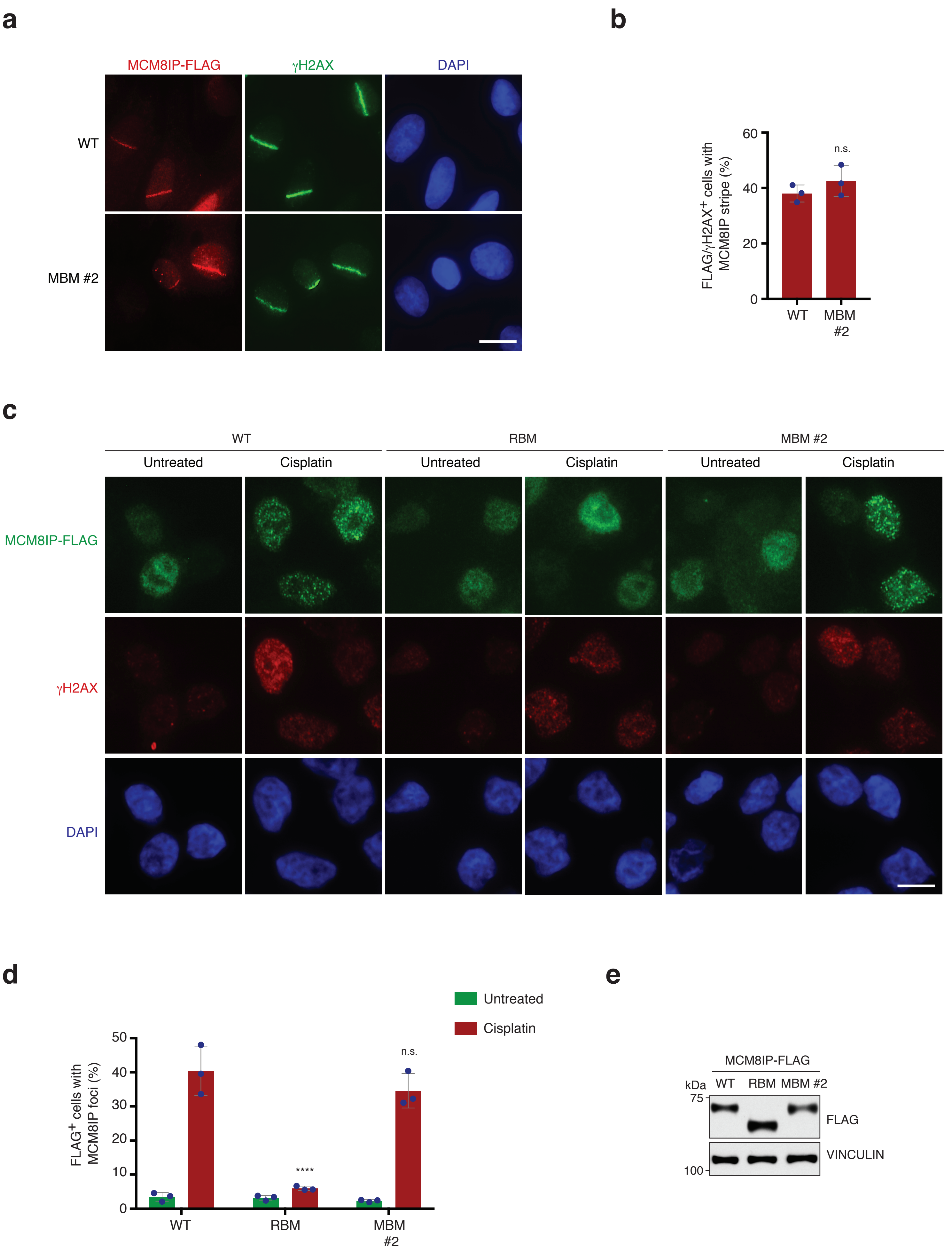

Supplementary Figure 4

Supplementary Figure 4. Characterization of the recruitment of MCM8IP to sites of DNA damage

a Representative images of the recruitment of MCM8IP-FLAG WT or MBM #2 in U2OS cells following UV laser microirradiation. DNA damage tracts are indicated with γH2AX staining. Scale bar = 20 μm.

b Graphical representation of the percentage of MCM8IP-FLAG WT or MBM #2 co-localizing with γH2AX following UV laser microirradiation in U2OS cells. The mean ± SD of three independent experiments is presented. Statistical analysis relative to MCM8IP-FLAG WT-expressing samples was conducted using Student's t-test (two-tailed).

c Representative images of MCM8IP nuclear foci in the HCT116 *MCM8IP* KO clone reconstituted with MCM8IP-FLAG WT, RBM or MBM #2 shown in **e** following treatment with 10 μM cisplatin for 24 hours. DNA damage is indicated with γH2AX staining. DAPI staining is also shown. Scale bar = 10 μm.

d Graphical representation of the percentage of MCM8IP-FLAG WT, RBM or MBM #2 foci-positive cells (≥5 foci) following cisplatin treatment, as described in **c**. The mean ± SD of three independent experiments is presented. Statistical analysis relative to WT-expressing cells after cisplatin was conducted by one-way ANOVA (****$p<0.0001$).

e Detection by western blot of MCM8IP in an HCT116 *MCM8IP* KO clone reconstituted with MCM8IP-FLAG WT, RBM or MBM #2. Vinculin is shown as a loading control.

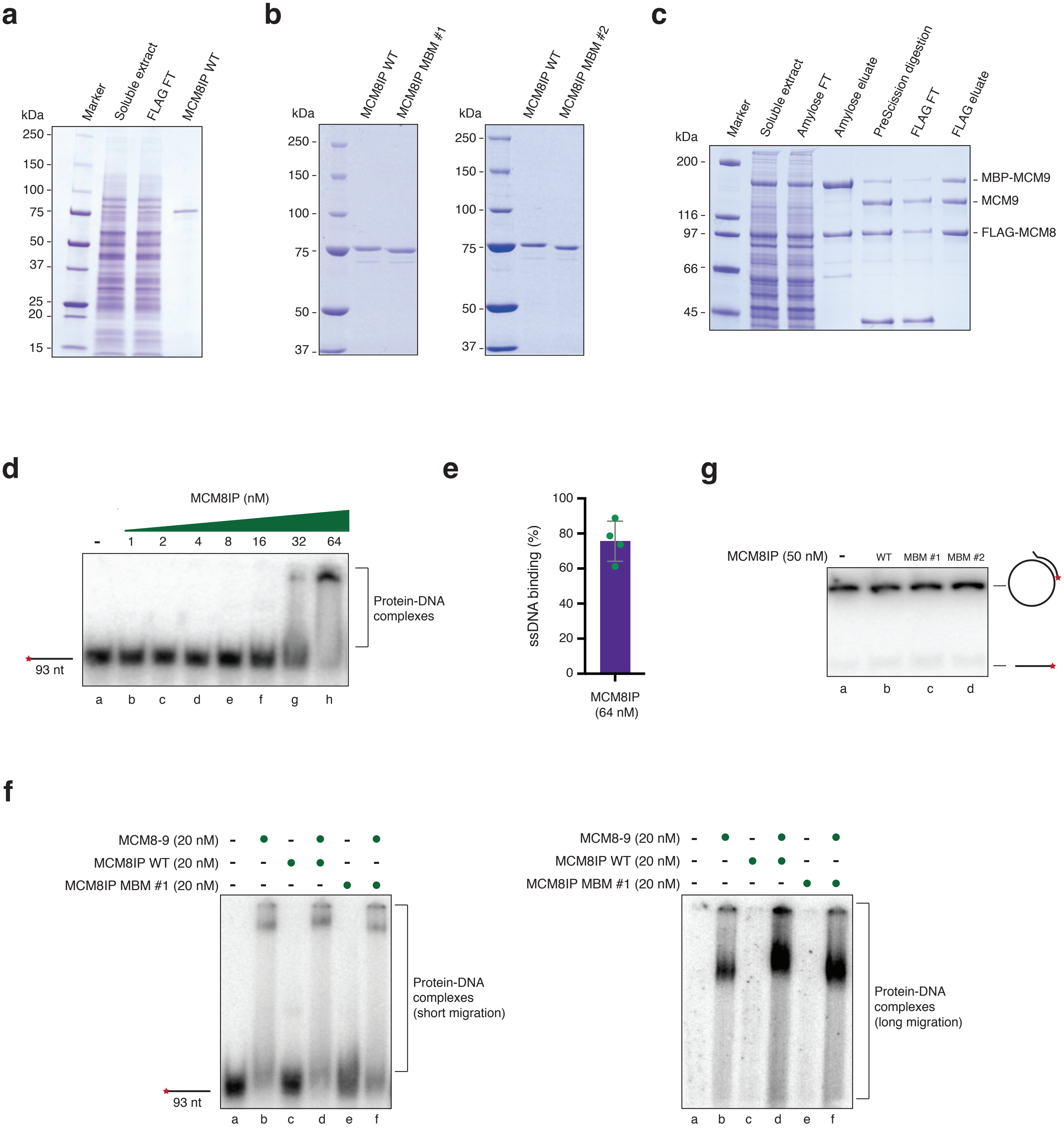

Supplementary Figure 5

Supplementary Figure 5. Characterization of ssDNA binding by MCM8IP and MCM8-9

a Coomassie-stained gel showing the purification of recombinant MCM8IP-FLAG WT from *Sf*9 cells. FLAG FT, flowthrough from the FLAG resin.

b Coomassie-stained gel showing purified MCM8IP-FLAG WT and MBM #1 (left panel) or MBM #2 proteins (right panel).

c Coomassie-stained gel showing the steps of the co-purification of recombinant MBP-MCM9 and FLAG-MCM8 from *Sf*9 cells. Amylose FT, flowthrough from the amylose resin; FLAG FT, flowthrough from the FLAG resin.

d Representative autoradiograph of an electrophoretic mobility shift assay with ^{32}P-labeled single-stranded 93-mer incubated with increasing amounts of recombinant MCM8IP.

e Graphical representation of the percentage of single-stranded 93-mer substrate bound by recombinant MCM8IP (64 nM) in electrophoretic mobility shift assays conducted as in **d**. The mean ± SD of four independent experiments is presented.

f Representative autoradiograph of an electrophoretic mobility shift assay with single-stranded 93-mer as in **d** incubated with recombinant MCM8-9 (20 nM), MCM8IP WT (20 nM) or MBM #1 (20 nM), either alone or in combination, as indicated (left panel). Longer migration of the electrophoretic mobility shift assay is shown in the right panel.

g Representative autoradiograph of an M13mp18-based DNA unwinding assay after incubation with 50 nM of purified WT MCM8IP, MBM #1 or MBM #2 proteins.

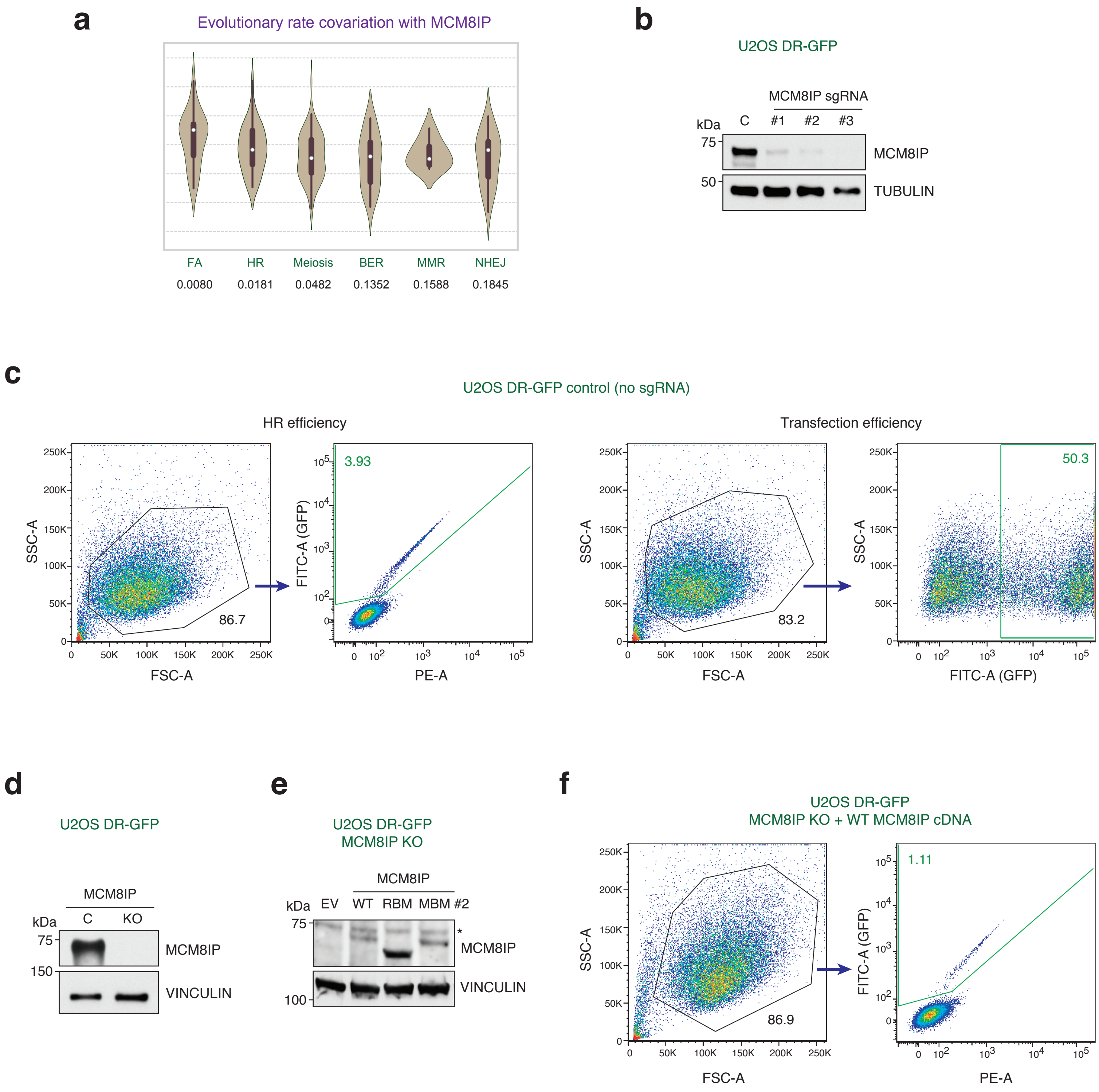

Supplementary Figure 6

Supplementary Figure 6. Characterization of the role of MCM8IP in homologous recombination

a Evolutionary Rate Covariation (ERC) analysis of MCM8IP with genes in different mammalian DNA repair pathways. Overlaid box plots indicate the quartiles of each distribution and a white dot represents the median. Permutation p-values listed below each DNA repair pathway reflect the significance of MCM8IP's co-evolution with each pathway. FA, Fanconi anemia; HR, homologous recombination; BER, base excision repair; MMR, mismatch repair; NHEJ, non-homologous end joining.

b Detection by western blot of MCM8IP in U2OS DR-GFP control cells or cells expressing the indicated MCM8IP sgRNAs. Tubulin is shown as a loading control.

c Gating strategy used to analyze fold changes in HR efficiency in U2OS DR-GFP cells, as presented in Figure **5b**. Gating of *I-SceI*-transfected U2OS DR-GFP non-targeting control cells is presented as an example. HR repair efficiency (left panel) was normalized to transfection efficiency, as determined in cells transfected with pEGFP-N3 (right panel), and expressed relative to the *I-SceI*-transfected non-targeting control.

d Detection by western blot of MCM8IP in U2OS DR-GFP control cells or an *MCM8IP* KO clone. Vinculin is shown as a loading control.

e Detection by western blot of MCM8IP in the U2OS DR-GFP *MCM8IP* KO clone shown in **d** upon reconstitution with WT MCM8IP, RBM, MBM #2 or an empty vector (EV) control. Vinculin is shown as a loading control. Asterisk indicates a non-specific band.

f Gating strategy used to analyze fold changes in HR efficiency in U2OS DR-GFP *MCM8IP* KO cells, as presented in Figure **5c**. Gating of HR repair events in *I-SceI*-transfected *MCM8IP* KO cells expressing WT MCM8IP is presented as an example. HR efficiency was normalized to transfection efficiency (determined as in **c**), and expressed relative to *I-SceI*-transfected *MCM8IP* KO cells complemented with WT MCM8IP.

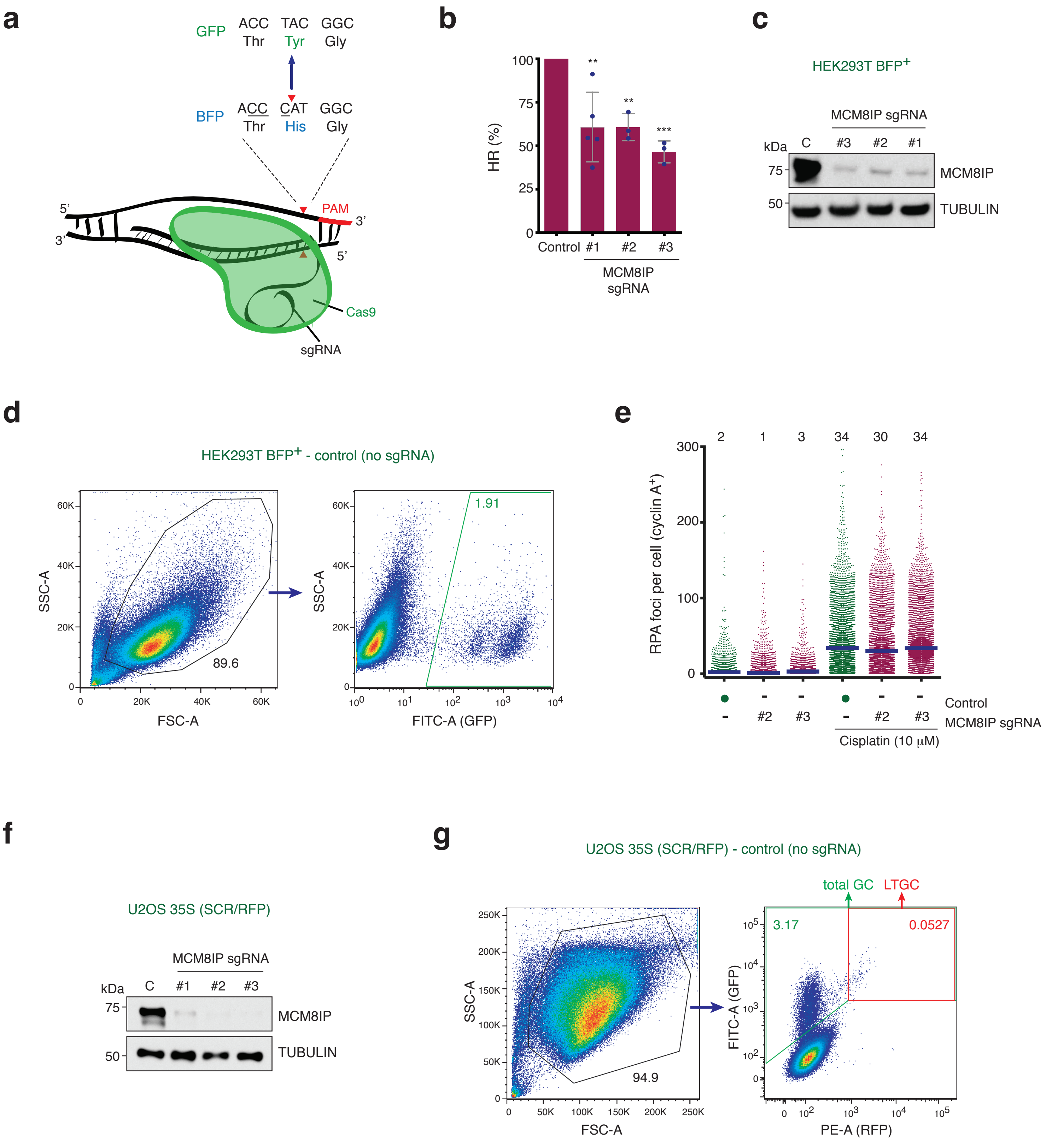

Supplementary Figure 7

Supplementary Figure 7. Analysis of the role of MCM8IP in the later stages of homologous recombination

a Schematic representation of the BFP gene conversion assay. Cas9 induces a DSB within the His66 codon of BFP (red arrowhead). Gene conversion results in Tyr66 and GFP expression.

b Graphical representation of the percentage of HR events in HEK293T BFP^+ cells expressing the indicated MCM8IP sgRNAs relative to control cells. The mean ± SD of three or more independent experiments (n = 3-5) is presented. Statistical analysis relative to control was conducted by one-way ANOVA (**$p<0.01$, ***$p<0.001$).

c Detection by western blot of MCM8IP in HEK293T BFP^+ control cells or cells expressing the indicated MCM8IP sgRNAs. Tubulin is shown as a loading control.

d Gating strategy used to determine fold changes in HR efficiency in HEK293T-BFP^+ cells, as presented in **b**. Gating of HR repair events in HEK293T-BFP^+ non-targeting control cells is shown as an example.

e Dot plot of the number of cisplatin-induced RPA2 foci in cyclin A-positive HCT116 control cells or cells expressing the indicated MCM8IP sgRNAs. Cells were fixed after 24 hours of cisplatin treatment (10 μM). RPA2 and cyclin A were stained for immunofluorescence, imaged by high-throughput microscopy and subjected to software-based quantitation. The median values are indicated above the dot plot and by blue lines within the dot plot. Data are representative of two independent experiments.

f Detection by western blot of MCM8IP in U2OS 35S control cells or cells expressing the indicated MCM8IP sgRNAs. Tubulin is shown as a loading control.

g Gating strategy used to analyze fold changes in STGC and LTGC efficiency and the ratio of LTGC/total GC in U2OS 35S cells, as presented in Figure **5e-f**. Gating of total GC events (GFP-positive/RFP-negative and RFP-positive) and LTGC events (GFP-positive/RFP-positive) in *I-*

SceI-transfected non-targeting control cells are presented as an example. STGC events (GFP-positive/RFP-negative) were determined by the subtraction of LTGC from total GC events. Repair efficiency was then normalized to transfection efficiency (determined as in Supplementary Figure **6c**), and expressed relative to the *I-SceI*-transfected non-targeting control.

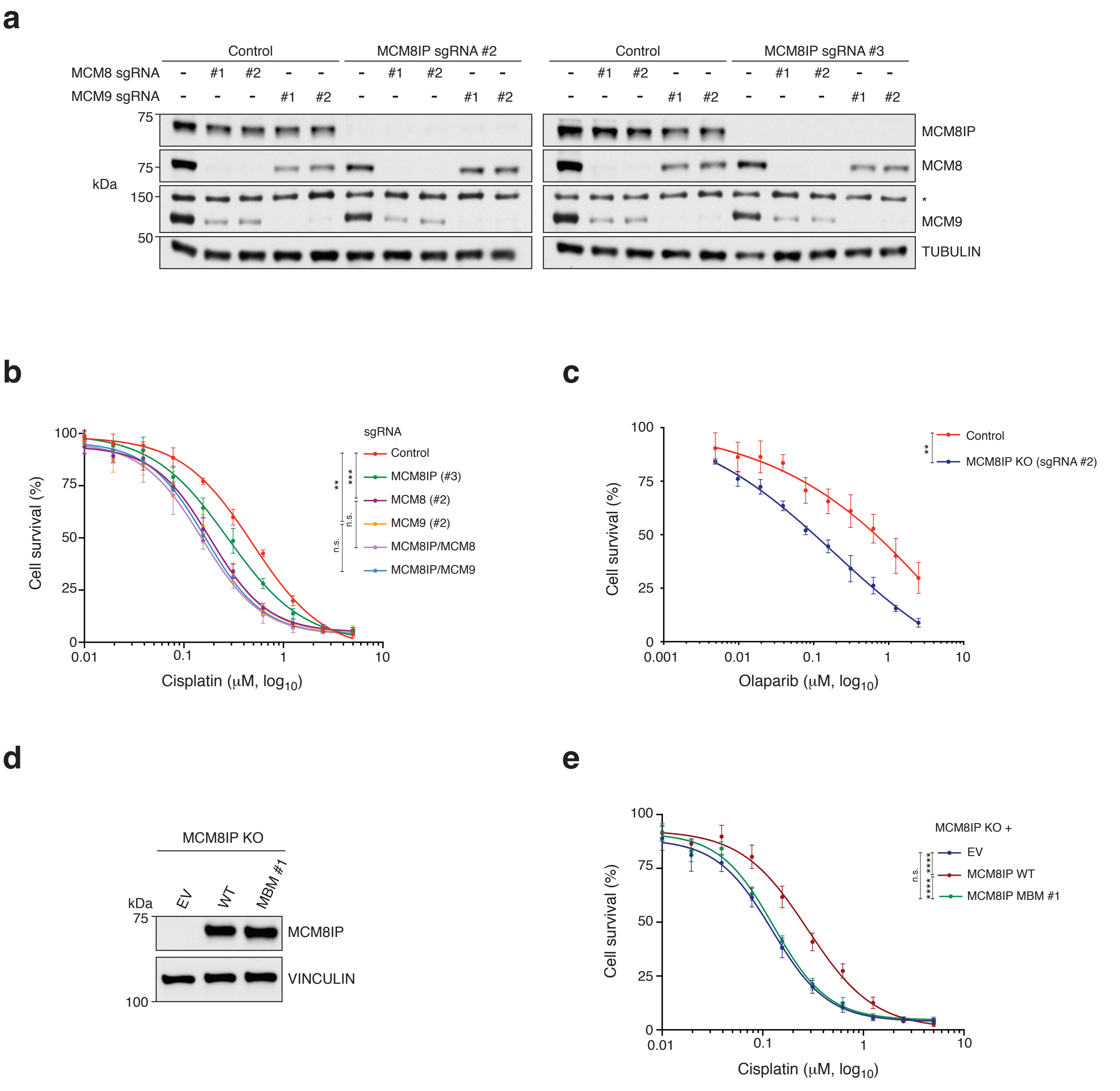

Supplementary Figure 8

Supplementary Figure 8. Survival analysis in MCM8IP-deficient cells upon cisplatin or olaparib treatment

a Detection by western blot of MCM8IP, MCM8 and MCM9 in HCT116 control cells or cells expressing the indicated sgRNAs. Tubulin is shown as a loading control. Asterisk indicates a non-specific band.

b Survival analysis of cisplatin-treated HCT116 control cells or cells expressing either MCM8IP sgRNA #3, MCM8 sgRNA #2 or MCM9 sgRNA #2 alone or in combination. Cell survival is expressed as a percentage of an untreated control. The mean ± SD of three or more independent experiments (n = 3-4) is presented. Statistical analysis was conducted using Student's t-test as in Figure **6a** (**$p<0.01$, ***$p<0.001$, at all three concentrations analyzed, two-tailed).

c Survival analysis of HCT116 control or *MCM8IP* KO cells in response to olaparib. Cell survival is expressed as a percentage of an untreated control. The mean ± SD of three independent experiments is presented. Statistical analysis was performed using Student's t-test as in Figure **6f** (**$p<0.01$, at all three concentrations analyzed, two-tailed).

d Detection of MCM8IP in HCT116 *MCM8IP* KO cells reconstituted with MCM8IP WT, MBM #1 or an empty vector (EV) control. Vinculin is shown as a loading control.

e Survival analysis of HCT116 *MCM8IP* KO cells reconstituted with EV, MCM8IP WT or MBM #1, as shown in **d**, in response to cisplatin. Cell survival is expressed as a percentage of an untreated control. The mean ± SD of seven independent experiments is presented. Statistical analysis was conducted using Student's t-test as in Figure **6a** (****$p<0.0001$, at all three concentrations analyzed, two-tailed).

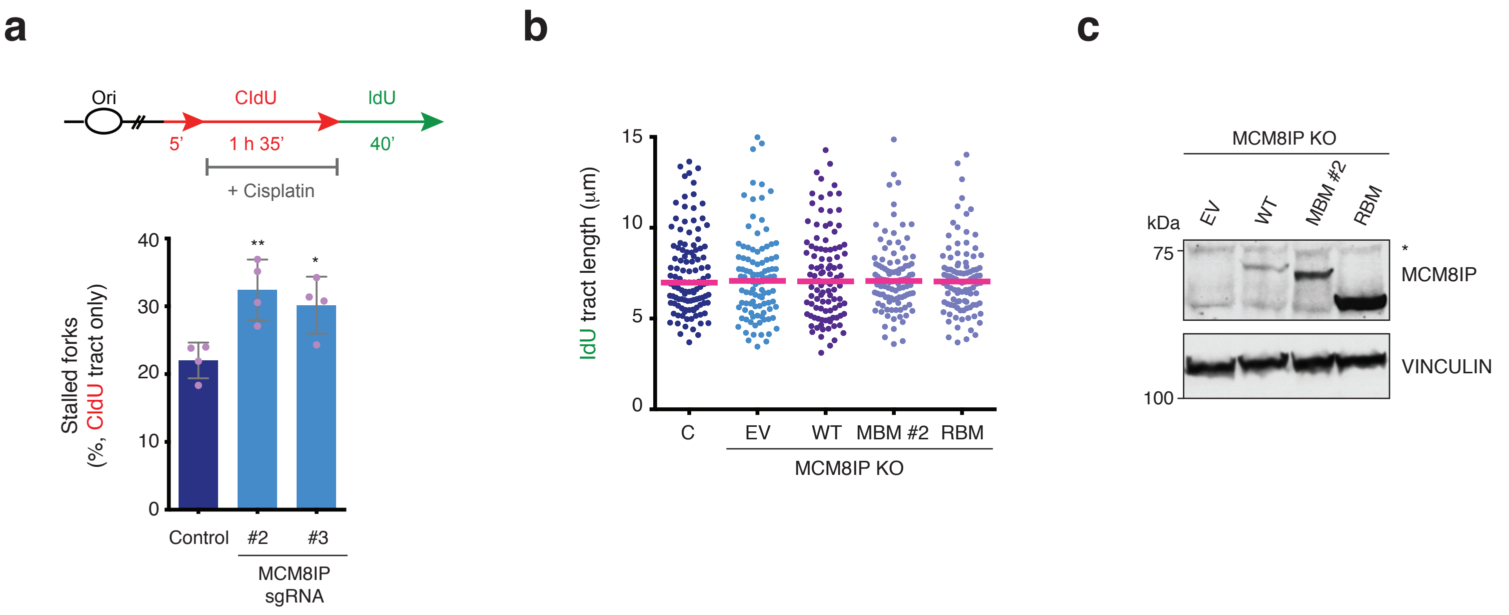

Supplementary Figure 9

Supplementary Figure 9. Characterization of the role of MCM8IP at replication forks in response to cisplatin treatment

a Schematic representation of a CldU/IdU pulse labeling assay (top panel) to assess the restart of stalled forks following treatment with cisplatin (30 μM). Graphical representation of the percentage of CldU-only tracts in cisplatin-treated HCT116 control cells or cells expressing the indicated MCM8IP sgRNAs (bottom panel). The mean ± SD of four independent experiments is presented. Statistical analysis relative to control was conducted by one-way ANOVA (*$p<0.05$, **$p<0.01$).

b Dot plot of IdU tract length for individual replication forks in untreated HCT116 control cells or *MCM8IP* KO cells reconstituted with MCM8IP WT, MBM #2, RBM or an empty vector (EV) control. Experiments were conducted as in Figure **7c**. Data are shown as in Figure **7b** and are representative of two independent experiments.

c Detection by western blot of MCM8IP in HCT116 *MCM8IP* KO cells reconstituted with MCM8IP WT, MBM #2, RBM or an EV control. Vinculin is shown as a loading control. Asterisk indicates a non-specific band.
