# Supplementary material for: MCM8IP activates the MCM8-9 helicase to promote DNA synthesis and homologous recombination upon DNA damage
Source: Nat Commun. 2020 Jun 11;11:2948. doi: 10.1038/s41467-020-16718-3 (PMC7290032; doi:10.1038/s41467-020-16718-3)
Supplement: Supplementary file 2 — Description of Additional Supplementary Files [file 41467_2020_16718_MOESM2_ESM.pdf]

Description of Additional Supplementary Files

File Name: Supplementary Data 1
Description: **List of proteins significantly enriched by BirA*-RPA1**
Proteins represented by 2 or more unique peptides and at least 3-fold enriched in total spectral counts (TSC) over the control, BirA* alone, are listed.

File Name: Supplementary Data 2
Description: **List of proteins significantly enriched in MCM8IP BioID and MCM8IP-HA immunoprecipitation experiments**
Proteins listed for BirA*-MCM8IP and MCM8IP-BirA* were represented by 2 or more unique peptides in each of two technical replicates and were at least 5-fold enriched in total spectral counts (TSC) over BirA* alone control (calculated from the average of two technical replicates). Similarly, proteins listed for MCM8IP-HA were represented by 2 or more unique peptides in each of two technical replicates and were at least 5-fold enriched in TSC over GFP-HA control (calculated from the average of two technical replicates).

File Name: Supplementary Data 3
Description: **List of MCM8, MCM9 and MCM8IP orthologs in 41 distinct species**
This table contains the RefSeq accessions for all MCM8, MCM9 and MCM8IP orthologs identified in BLAST searches. A “0” indicates that no ortholog was found. A “+” sign indicates the presence of the gene in the indicated species, as previously reported[1]. This table was utilized to build the phylogenetic tree of Supplementary Figure **3d**.

File Name: Supplementary Data 4
Description: **Phylogenetic studies of correlated gene loss for *MCM8*, *MCM9* and *MCM8IP***
This table contains the results of the BayesTraits runs for all three paired combinations of the *MCM8*, *MCM9*, and *MCM8IP* genes. Independent and dependent model parameters (gene loss rates) are reported from the maximum likelihood models. Root parameters reflect the state of the genes at the root of the tree. Since gene gain was not allowed, it follows that the probability of both genes being present at the base of the tree, P(1,1), is 1. Log likelihood of each model and the likelihood ratio tests between them are reported to test the rejection of the independent model in favor of the dependent model. P-values were estimated with the chi-square distribution with 2 degrees of freedom.

File Name: Supplementary Data 5
Description: **Primer sequences used in this study**
This table lists the primers used to generate the MCM8IP, MCM8 and MCM9 constructs used in this study. Sequence information and a description, including references to corresponding figures, are provided. Asterisks indicate primers that are used more than once.

SUPPLEMENTARY REFERENCE

1. Liu, Y., Richards, T.A. & Aves, S.J. Ancient diversification of eukaryotic MCM DNA replication proteins. *BMC Evol Biol* **9**, 60 (2009).
