# Supplementary material for: MCM8IP activates the MCM8-9 helicase to promote DNA synthesis and homologous recombination upon DNA damage
Source: Nat Commun. 2020 Jun 11;11:2948. doi: 10.1038/s41467-020-16718-3 (PMC7290032; doi:10.1038/s41467-020-16718-3)
Supplement: Supplementary file 8 — Reporting Summary [file 41467_2020_16718_MOESM8_ESM.pdf]

nature research

Corresponding author(s): Alberto Ciccia

Last updated by author(s): 5/15/2020

Reporting Summary

Nature Research wishes to improve the reproducibility of the work that we publish. This form provides structure for consistency and transparency in reporting. For further information on Nature Research policies, see Authors & Referees and the Editorial Policy Checklist.

Statistics

For all statistical analyses, confirm that the following items are present in the figure legend, table legend, main text, or Methods section.

n/a	Confirmed	
☐	☒	The exact sample size (*n*) for each experimental group/condition, given as a discrete number and unit of measurement
☐	☒	A statement on whether measurements were taken from distinct samples or whether the same sample was measured repeatedly
☐	☒	The statistical test(s) used AND whether they are one- or two-sided<br>*Only common tests should be described solely by name; describe more complex techniques in the Methods section.*
☒	☐	A description of all covariates tested
☒	☐	A description of any assumptions or corrections, such as tests of normality and adjustment for multiple comparisons
☐	☒	A full description of the statistical parameters including central tendency (e.g. means) or other basic estimates (e.g. regression coefficient) AND variation (e.g. standard deviation) or associated estimates of uncertainty (e.g. confidence intervals)
☐	☒	For null hypothesis testing, the test statistic (e.g. *F*, *t*, *r*) with confidence intervals, effect sizes, degrees of freedom and *P* value noted<br>*Give P values as exact values whenever suitable.*
☐	☒	For Bayesian analysis, information on the choice of priors and Markov chain Monte Carlo settings
☒	☐	For hierarchical and complex designs, identification of the appropriate level for tests and full reporting of outcomes
☒	☐	Estimates of effect sizes (e.g. Cohen's *d*, Pearson's *r*), indicating how they were calculated

Our web collection on statistics for biologists contains articles on many of the points above.

Software and code

Policy information about availability of computer code

Data collection	Q Exactive HF and Orbitrap Fusion Tribrid mass spectrometers, BD LSRFortessa and BD LSRII flow cytometers operated with BD FACSDiva 8.0 software, Zeiss AxioObserver.Z1 with PALM MicroBeam IV micro-dissection microscope operated with PALMRobo 4.6 software, Typhoon Phosphor Imager (FLA 9500, Version 1.0, Build 1.0.0.185), InGenius3 geldoc imager operated with InGenius3 GENESys v1.5.9.0 software, ImageXpress Nano high-content microscope operated with MetaXpress 6 software, Nikon Eclipse 90i microscope operated with NIS Elements BR 5.02.01 software, SpectraMax ID3 spectrophotometer operated with Softmax Pro 7.0.3 software, Epson V600 Photo scanner operated with Epson Scan v3.9.4.0 US software, CanoScan 9000F Mark II scanner operated with ImageCapture v6.6(525) software, Microsoft Excel
Data analysis	GraphPad Prism v6, FlowJo v9.9.5, online tools Clustal Omega and ESPript 3.0, BayesTraits v2, Fiji v2.0.0-rc-69, ImageJ v1.52p, MetaXpress 6, MaxQuant v1.6.1.0, Scaffold 4

For manuscripts utilizing custom algorithms or software that are central to the research but not yet described in published literature, software must be made available to editors/reviewers. We strongly encourage code deposition in a community repository (e.g. GitHub). See the Nature Research guidelines for submitting code & software for further information.

Data

Policy information about availability of data

All manuscripts must include a data availability statement. This statement should provide the following information, where applicable:

- Accession codes, unique identifiers, or web links for publicly available datasets
- A list of figures that have associated raw data
- A description of any restrictions on data availability

The datasets generated during and/or analyzed during the current study are available from the corresponding author upon reasonable request. Mass spectrometry datasets are deposited in PRIDE database (Accession number: PXD019156). The source data underlying Figures 1c-e, g, 2a, c-d, f, 3b, d, f, 4a-f, 5b-d, f, 6a-i, and 7b, d, f and Supplementary Figures 1b-d, 2b, 3a-c, 4b, d, e, 5a-g, 6a-b, d-e, 7b-c, e-f, 8a-e, and 9a-b are provided as a Source Data file.

Field-specific reporting

Please select the one below that is the best fit for your research. If you are not sure, read the appropriate sections before making your selection.

☒ Life sciences ☐ Behavioural & social sciences ☐ Ecological, evolutionary & environmental sciences

For a reference copy of the document with all sections, see nature.com/documents/nr-reporting-summary-flat.pdf

Life sciences study design

All studies must disclose on these points even when the disclosure is negative.

Sample size

No statistical method was used to predetermine sample size. The samples sizes selected for quantitative studies are based on previous studies in the field with similar methodologies (e.g., Daza-Martin et al., PMID: 31270457; Noordermeer et al., PMID: 30022168; Adelman et al., PMID: 24005329) and sample numbers are of appropriate size for analyses of statistical significance.

Data exclusions

No data have been excluded.

Replication

Cell-based assays (cell survival, homologous recombination assays, MCM8IP localization) and biochemical reactions (EMSA and helicase assays) were reproduced in at least 3 independent biological replicates. Western blotting, immunoprecipitation and pulldown assays are representative of at least 2 independent experiments each. The DNA fiber and RAD51 foci experiments are representative of at least 2 biological replicates. All attempts at replication were successful with quantitative data analyzed for statistical significance.

Randomization

There was no randomization in this study as the nature of our experiments did not require them.

Blinding

Data from survival assays, HR repair assays, RAD51 and RPA foci assays, and EMSA and helicase assays were collected and analyzed digitally with consistent settings, and therefore did not require blinding. Blinding was not feasible for western blotting and interaction studies and would unlikely influence outcome. DNA fiber assays and MCM8IP-FLAG localization assays (foci and UV-damaged tract formation) were not blinded as they were stained with commercially available and widely-used antibodies that yielded robust signals for scoring by eye. Additionally, they were conducted appropriately with respect to experimental setup (i.e., untreated controls), data collection (i.e., microscopic fields chosen at random and imaged with the same settings) and data analysis (i.e., scoring under the same image settings). Following our analyses, the phenotypes were apparent and we are therefore confident that blinding would not have yielded meaningfully different outcomes.

Reporting for specific materials, systems and methods

We require information from authors about some types of materials, experimental systems and methods used in many studies. Here, indicate whether each material, system or method listed is relevant to your study. If you are not sure if a list item applies to your research, read the appropriate section before selecting a response.

Materials & experimental systems

n/a	Involved in the study
☐	☒ Antibodies
☐	☒ Eukaryotic cell lines
☒	☐ Palaeontology
☒	☐ Animals and other organisms
☒	☐ Human research participants
☒	☐ Clinical data

Methods

n/a	Involved in the study
☒	☐ ChIP-seq
☐	☒ Flow cytometry
☒	☐ MRI-based neuroimaging

Antibodies

Antibodies used

The antibodies used in this study for immunofluorescence/immunocytochemistry (IF) and western blotting (WB) are as follows: rabbit anti-C17orf53 (Sigma HPA023393) (WB), mouse anti-C17orf53 (Novus NBP2-37407) (WB), rabbit anti-MCM8 (Proteintech 16451-1-AP) (WB), rabbit anti-MCM9 (Millipore ABE2603) (WB), rabbit anti-RPA1 (Bethyl A300-241A) (WB), rabbit anti-RPA2 (Bethyl A300-244A) (IF and WB), rabbit anti-SMARCAL1 (Bethyl A301-616A) (WB), mouse anti-SMARCAL1 (Santa Cruz sc-376377) (WB), rabbit anti-CtIP (Bethyl A300-488A) (WB), rabbit anti-GST (Abcam ab21070) (WB), mouse anti-GST (Santa Cruz sc-138) (WB), mouse anti-FLAG (Sigma F1804) (IF and WB), mouse anti-HA (Sigma H3663) (WB), rat anti-tubulin (Novus NB600-506) (WB), mouse anti-vinculin (Sigma V9131) (WB), rabbit anti-Lamin B1 (ThermoFisher PA5-19468) (WB), rabbit anti-histone H3 (Bethyl A300-823A) (WB), rabbit anti-RAD51 (BioAcademia BAM-70-002) (IF), mouse anti-cyclin A (Santa Cruz sc-271682)(IF), rabbit anti-γH2AX (Bethyl A300-081A)(IF), rat anti-BrdU (Abcam ab6326)(IF), and mouse anti-BrdU (BD Biosciences 347580)(IF). Working dilutions are indicated in the Methods section.

Validation

The antibodies used in this study are available commercially with specificity and application data available online. Applications in our study for each antibody are listed in the previous section and in the manuscript. In particular, the specificities of C17orf53 (both anti-rabbit and anti-mouse), MCM8 and MCM9 antibodies were each validated with some combination of sgRNA-mediated

deletion, siRNA-induced depletion, and/or cDNA (wildtype and mutant) overexpression studies in our cell lines by western blotting and/or immunoprecipitation, many of which are presented in the manuscript. Furthermore, specificity of the MCM9 antibody was previously reported (Park et al, PMID: 23401855).

Eukaryotic cell lines

Policy information about cell lines

Cell line source(s)	U2OS, HEK293T and HCT116 were obtained from ATCC. HEK293T T-REx cells were obtained from Life Technologies/Thermo Fisher. U2OS DR-GFP was gifted by Maria Jasin's lab (Memorial Sloan Kettering Cancer Center, New York, NY). U2OS 35S cells were gifted by Ralph Scully's lab (Harvard Medical School, Boston, MA).
Authentication	Cell lines were not independently authenticated.
Mycoplasma contamination	Cell lines were not independently tested for mycoplasma contamination.
Commonly misidentified lines (See ICLAC register)	No commonly misidentified cell lines were used in this study (ICLAC, v10).

Flow Cytometry

Plots

Confirm that:

☒ The axis labels state the marker and fluorochrome used (e.g. CD4-FITC).

☒ The axis scales are clearly visible. Include numbers along axes only for bottom left plot of group (a 'group' is an analysis of identical markers).

☒ All plots are contour plots with outliers or pseudocolor plots.

☒ A numerical value for number of cells or percentage (with statistics) is provided.

Methodology

Sample preparation

U2OS DR-GFP, U2OS DR-GFP-derived MCM8IP-KO clone and U2OS 35S (SCR/RFP) cells were seeded in 6-well tissue culture plates so as to achieve 60-70% confluency the following day. Cells were then transfected with 2.5 µg of an I-SceI expression vector or pEGFP-N3. In parallel experiments, control cells (non-targeting or empty vector-complemented KO cells) were transfected with 2.5 µg of pCAGGS (empty vector control) to determine GFP/RFP-positivity. Two days after transfection, cells were trypsinized, washed once in PBS, and subjected to flow cytometry.

HEK293T-BFP+ cells were seeded in 24-well tissue culture plates so as to achieve 50-70% confluency the following day. Cells were then co-transfected with 250 ng of pX330 (SpCas9-expressing plasmid containing a BFP-targeting sgRNA) and 500 ng of plasmid containing an HDR donor sequence. Three days after transfection, cells were trypsinized, washed once in PBS, and subjected to flow cytometry.

Instrument	BD LSRII (DR-GFP assay) or BD LSRFortessa (SCR/RFP assay and BFP conversion assay)
Software	BD FACSDiva Software 8.0
Cell population abundance	No post-sort fractions were collected.

Gating strategy

For U2OS DR-GFP, U2OS DR-GFP-derived MCM8IP-KO clone and U2OS 35S (SCR/RFP) cells, FSC/SSC parameters were initially used to gate the majority of the population to the exclusion of cell debris. From that gated population, FITC-A/PE-A parameters were then used to determine GFP- and/or RFP-positivity (HR repair events) in the DR-GFP and SCR-RFP assays. Discerning positivity from negativity was determined by initially positioning gates in pCAGGS-transfected (negative control) and I-SceI-transfected (positive control) non-targeting or empty vector-complemented KO cells. Once established, the same gates were then applied to MCM8IP sgRNA-expressing or MCM8IP cDNA-complemented KO cells. A similar gating strategy for determining GFP-positivity in our transfection efficiency controls (pEGFP-N3-transfected cells) was used but with SSC-A/FITC-A parameters.

For HEK239T-BFP+ cells, FSC/SSC parameters were initially used to gate the majority of the population to the exclusion of cell debris. From that gated population, SSC-A/FITC-A parameters were then used to determine GFP-positivity (HR repair events) in the BFP conversion assay. Discerning positivity from negativity was determined by initially positioning gates in pX330-BFP sgRNA-transfected (negative control) control cells and cells co-transfected with px330-BFP sgRNA and an HDR donor (positive control). Once established, the same gates were then applied to MCM8IP sgRNA-expressing cells.

☒ Tick this box to confirm that a figure exemplifying the gating strategy is provided in the Supplementary Information.
